# The machinery underlying malaria parasite virulence is conserved between rodent and human malaria parasites

**DOI:** 10.1038/ncomms11659

**Published:** 2016-05-26

**Authors:** Mariana De Niz, Ann-Katrin Ullrich, Arlett Heiber, Alexandra Blancke Soares, Christian Pick, Ruth Lyck, Derya Keller, Gesine Kaiser, Monica Prado, Sven Flemming, Hernando del Portillo, Chris J. Janse, Volker Heussler, Tobias Spielmann

**Affiliations:** 1 Institute of Cell Biology, University of Bern, 3012 Bern, Switzerland; 2 Graduate School for Cellular and Biomedical Sciences, University of Bern, Bern 3012, Switzerland; 3 Bernhard Nocht Institute for Tropical Medicine, Parasitology Section, 20359 Hamburg, Germany; 4 Institute of Zoology, Universtity of Hamburg, 20146 Hamburg, Germany; 5 Theodor Kocher Institute, University of Bern, 3012 Bern, Switzerland; 6 ICREA at ISGlobal, Barcelona Ctr. Int. Health Res. (CRESIB), Hospital Clínic—Universitat de Barcelona and Institut d'Investigació Germans Trias i Pujol (IGTP). Ctra. de Can Ruti. Camí de les Escoles, s/n, 08916 Badalona, Spain; 7 Leiden Malaria Research Group, Department of Parasitology, Leiden University Medical Center, 2333 ZA Leiden, The Netherlands

## Abstract

Sequestration of red blood cells infected with the human malaria parasite *Plasmodium falciparum* in organs such as the brain is considered important for pathogenicity. A similar phenomenon has been observed in mouse models of malaria, using the rodent parasite *Plasmodium berghei*, but it is unclear whether the *P. falciparum* proteins known to be involved in this process are conserved in the rodent parasite. Here we identify the *P. berghei* orthologues of two such key factors of *P. falciparum*, SBP1 and MAHRP1. Red blood cells infected with *P. berghei* parasites lacking SBP1 or MAHRP1a fail to bind the endothelial receptor CD36 and show reduced sequestration and virulence in mice. Complementation of the mutant *P. berghei* parasites with the respective *P. falciparum* SBP1 and MAHRP1 orthologues restores sequestration and virulence. These findings reveal evolutionary conservation of the machinery underlying sequestration of divergent malaria parasites and support the notion that the *P. berghei* rodent model is an adequate tool for research on malaria virulence.

## Abstract (web summary)

Proteins SBP1 and MAHRP1 of the human malaria parasite are required for sequestration of infected red blood cells in major organs. Here, De Niz *et al*. identify homologous proteins in the rodent parasite *Plasmodium berghei*, showing that they play similar roles and supporting the usefulness of malaria mouse models.

## Graphical abstract

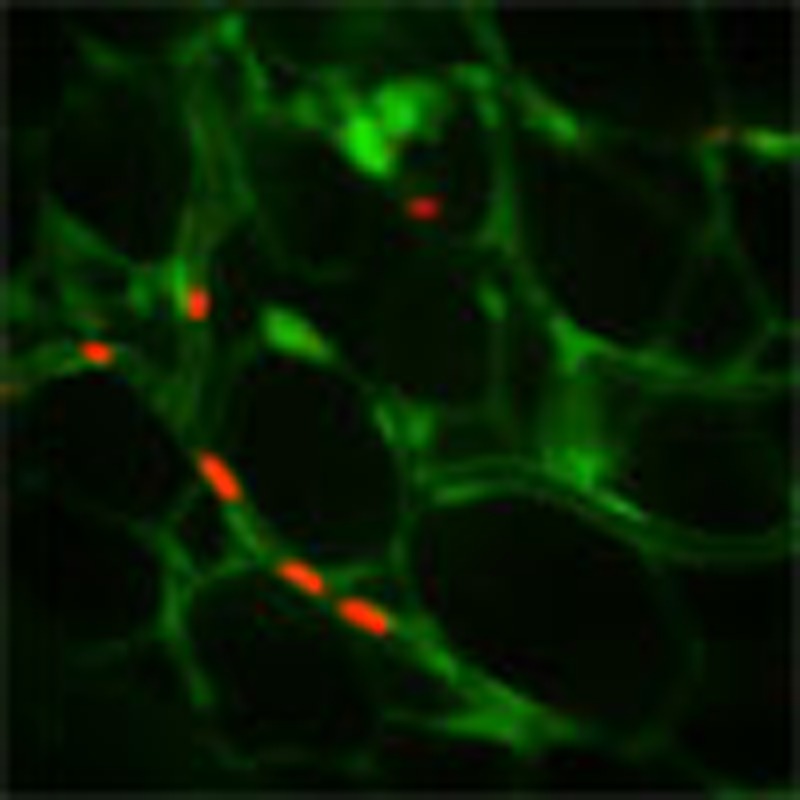

A hallmark of *Plasmodium falciparum* malaria is the adhesion of infected red blood cells (iRBCs) to the endothelium of capillary venules, leading to sequestration in multiple organs of the human host^1^,^2^. Sequestration is associated with life-threatening complications and considered to be central to the severe pathogenesis of this parasite^3^. Sequestration of *P. falciparum* parasites is mediated by the major virulence factor PfEMP1, a protein transported to the iRBC surface that enables binding to endothelial host cell receptors such as CD36 and ICAM1 (ref. 4). How sequestration of iRBCs contributes to pathology is not fully understood and difficult to study in humans^5^. In rodent malaria models, which conveniently allow *in vivo* virulence studies^6^,^7^,^8^,^9^,^10^, sequestration occurs^11^,^12^ but PfEMP1 homologues are absent^13^.

For PfEMP1 to reach the iRBC cytosol, it first traverses the parasitophorous vacuole membrane (PVM) using a putative translocon of exported proteins that is conserved among different *Plasmodium* species and involved in transport of all exported proteins^14^,^15^,^16^. In the host cell an elaborate machinery then mediates further transport of PfEMP1 to the iRBC surface and the proper surface display required for cytoadherence^17^,^18^,^19^,^20^. This machinery was so far considered to be specific for the transport of PfEMP1 in *P. falciparum* and does not seem to be required for the trafficking of other exported proteins^17^,^18^,^19^,^20^. This PfEMP1-specific transport machinery was proposed as one possible reason for the expansion of the number of exported proteins seen in *P. falciparum*^21^.

Two molecules of this trafficking machinery previously shown to be essential for the transport of PfEMP1 to the RBC surface are SBP1 (ref. 22) and MAHRP1 (ref. 23). Both proteins belong to the PEXEL-negative exported proteins (PNEPs)^24^ that lack the PEXEL motif found in many exported proteins^25^. Parasites with genetic ablations of either *sbp1* or *mahrp1* failed to cytoadhere to receptors and endothelial cells *in vitro*^17^,^18^,^20^. In line with the absence of PfEMP1 in rodent malarias and the notion of a PfEMP1-specific transport machinery in *P. falciparum* iRBCs, neither SBP1 nor MAHRP1 had previously been identified in rodent malaria parasites. In fact, to our knowledge no PNEPs had been reported that were conserved between *P. falciparum* and rodent malarias. The only protein so far shown to be essential for *P. berghei* CD36-mediated sequestration of schizont-infected RBC is a PEXEL-positive exported protein, the schizont membrane-associated cytoadherence protein (SMAC), a molecule restricted to rodent malaria parasites that is found in the cytoplasm of iRBCs but not on their surface^8^.

Here we show that despite the absence of PfEMP1, *P. berghei* expresses orthologues of both PfSBP1 and PfMAHRP1 that are exported into the cytoplasm of iRBCs and are required for the transport of a still unidentified parasite ligand that allows binding of iRBCs to CD36 *in vitro* and sequestration *in vivo*. Strikingly, the *P. falciparum* orthologues PfSBP1 and PfMAHRP1 complement the respective gene deletions in *P. berghei.* Our data indicate evolutionary conservation of the machinery underlying parasite virulence and highlight the *P. berghei* rodent model as an adequate tool to analyse factors involved in malaria virulence.

## Results

### Orthologues of PfSBP1 and PfMAHRP1

**Figure 1:**
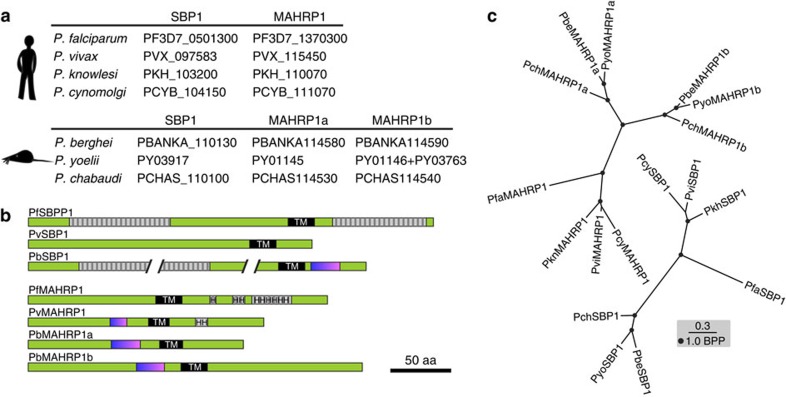
Orthologues of PfSBP1 and PfMAHRP1 in different *Plasmodium* species. (**a**) PlasmoDB IDs of the identified SBP1 and MAHRP1 orthologues. Top, human and primate infecting *Plasmodium* species; bottom, rodent-infecting *Plasmodium* species. (**b**) Domain structure of selected SBP1 and MAHRP1 orthologues. Hatched grey boxes show repeats; H, histidine-rich regions; black, transmembrane domains (TM); blue/magenta, oligo E/D-K/R stretches. The scale is 50 amino acids (aa). (**c**) Phylogenetic trees of the PfSBP1 and PfMAHRP1 orthologues. Scale bar indicates 0.3 expected substitutions per site; BBP, Bayesian posterior probability.

Using amino-acid similarity searches we detected putative PfSBP1 and PfMAHRP1 orthologues in all *Plasmodium* species listed in PlasmoDB (www.Plasmodb.org) (Fig. 1a; Supplementary Fig. 1a). Rodent *Plasmodium* species appear to possess two MAHRP1 versions that we termed MAHRP1a and MAHRP1b, and are encoded by tandem genes on the same locus. While the overall amino-acid similarity between the SBP1 and MAHRP1 homologues was rather low (Supplementary Fig. 1b–d), the architecture of the protein features was similar (Fig. 1b) and all lack a PEXEL motif. In addition, three other findings indicated that these proteins are indeed orthologues. Firstly, the phylogenetic trees of these proteins (Fig. 1c) are topologically concordant with the species tree of malaria parasites^26^; secondly, a jackhmmer search arrived at the same proteins originally detected by our similarity searches (Supplementary Fig. 2); and finally, a re-examination of the genomic location revealed that the genes encoding the MAHRP1 and SBP1 orthologues are in fact syntenic (Supplementary Fig. 3). The synteny to the corresponding *P. falciparum* genes has previously not been detected probably because it is obscured by neighbouring synteny breaks.

### Location and trafficking of SBP1 and MAHRP1 are conserved

**Figure 2:**
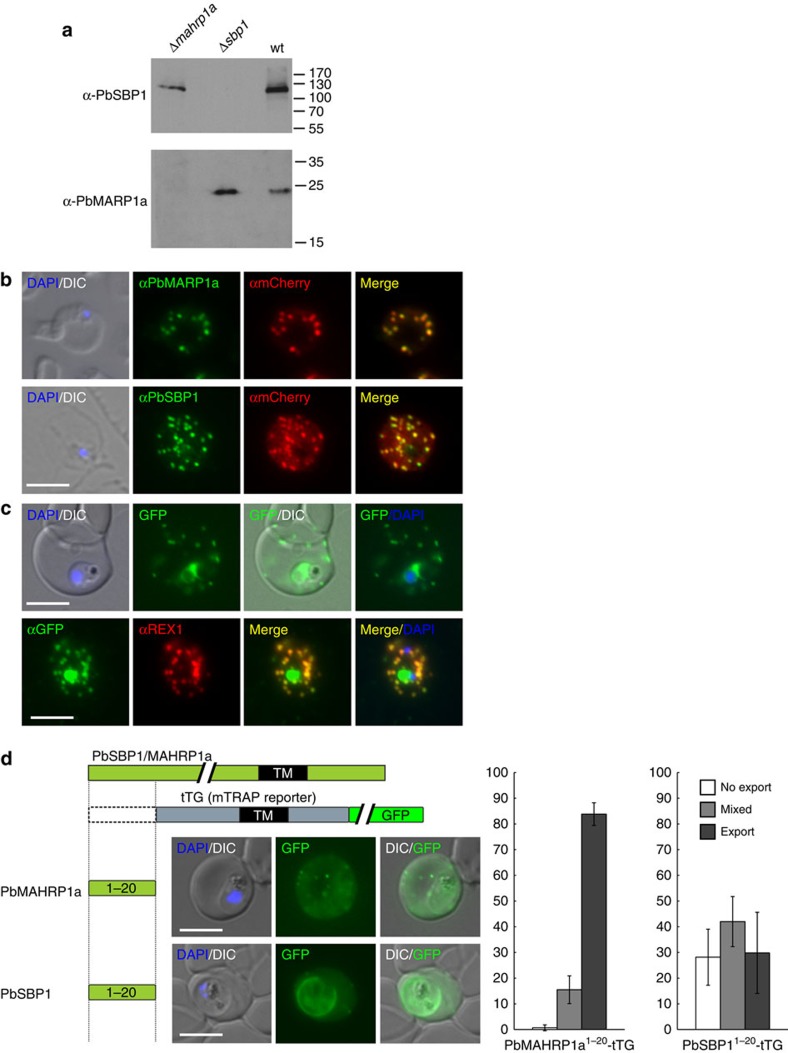
PbSBP1 and PbMAHRP1a localize to Maurer's clefts-like structures in *P. berghei-*infected erythrocytes and are trafficked by conserved export domains. (**a**) Immunoblot with specific antisera showing expression of the targeted proteins in blood stages of wild-type (wt) parasites and absence of expression in the respective gene-deletion mutants. Top (probed with an anti-PbSBP1 antiserum) and bottom (probed with an anti-PbMAHRP1a antiserum) show the same samples run on different gels and either PbSBP1 or PbMAHRP1a serve as loading control for the gene-deletion parasite extracts. The uncropped blots are shown in Supplementary Fig. 4. (**b**) IFA analysis of the localization of PbSBP1 and PbMAHRP1a (detected with specific sera) in IBIS1-mCherry expressing *P. berghei* parasites (detected using anti-mCherry antibodies) show co-localization in the cytosol of iRBC with mCherry-tagged IBIS. (**c**) PbSBP1 fused to GFP and expressed in *P. falciparum* blood stages is found at the Maurer's clefts. Top panel, live *P. falciparum* parasites expressing PbSBP1-GFP show punctate fluorescence in the RBC cytosol, typical for staining of Maurer's clefts. The perinuclear signal indicates partial retention of PbSBP1-GFP in the ER. Bottom panel, IFA with these parasites shows co-localization of PbSBP1-GFP with the Maurer's clefts marker protein REX1^30^. (**d**) The first 20 amino acids of either PbSBP1 or PbMAHRP1a promote export of a GFP-containing reporter protein (tTG)^31^ in *P. falciparum* iRBCs. Left: representative images of live blood stage parasites. Right: quantification of export determined by counting the number of cells (%) showing no export (staining in the PV and within the parasite), mixed export (staining in the PV/parasite and in the host cell) and full export (signal in the host cell only)^31^. Graphs show the mean of blinded scoring of at least 50 cells on each of *n*=3 occasions. Error bars represent s.d. (**c**,**d**), DIC, differential interference contrast; merge, merge of red and green channel; size bars: 5 μm.

In *P. falciparum*, SBP1 and MAHRP1 are exported into the iRBC cytoplasm where they are located at parasite-induced host cell modifications termed Maurer's clefts. These structures are believed to be important for the sorting of parasite proteins in the iRBC, including the transport of the virulence factor PfEMP1 (ref. 27). To analyse the location in iRBC of the *P. berghei* orthologues PbSBP1 (PBANKA_110130) and PbMAHRP1a (PBANKA114580) (MAHRP1b (PBANKA114590) was not further analysed, as a GFP fusion was poorly expressed in *P. berghei* blood stages), we raised specific antisera against these proteins (Fig. 2a; Supplementary Fig. 4). Immunofluorescence assays with these sera showed that PbSBP1 and PbMAHRP1a were exported into *P. berghei* iRBCs where they were found in multiple discrete foci (Fig. 2b; Supplementary Movie 1), resembling the typical labelling of proteins located in *P. falciparum* Maurer's clefts. In agreement with such a localization, both PbSBP1 and PbMAHRP1a co-localized with mCherry-tagged IBIS1 (Fig. 2b), a marker protein of previously identified Maurer's clefts-like structures in *P. berghei*^28^. Consistent with previous work^28^,^29^, these PbSBP1-positive structures were highly mobile in the cytosol of *P. berghei* iRBCs (Supplementary Movie 2). When *P. berghei* SBP1 was expressed as a GFP-fusion protein in *P. falciparum* blood stages, PbSBP1-GFP was detected in Maurer's clefts together with the *P. falciparum* Maurer's clefts marker REX1 (ref. 30; Fig. 2c), further supporting a localization at equivalent structures in both parasite species. Furthermore, the first 20 amino acids of PbMAHRP1a and PbSBP1, a region mediating export in *P. falciparum* PNEPs^31^, were sufficient to export an established reporter protein in *P. falciparum* into the host cell (Fig. 2d), indicating evolutionary conservation of the export signals in these orthologues and in PNEPs of different *Plasmodium* species in general. Taken together these data establish that SBP1 and MAHRP1 represent the first known Maurer's clefts proteins conserved across all *Plasmodium* species analysed so far, and they are amongst only a handful of conserved exported proteins discovered to date^21^,^26^,^32^.

### PbSBP1 and PbMAHRP1a are required for iRBC sequestration

**Figure 3:**
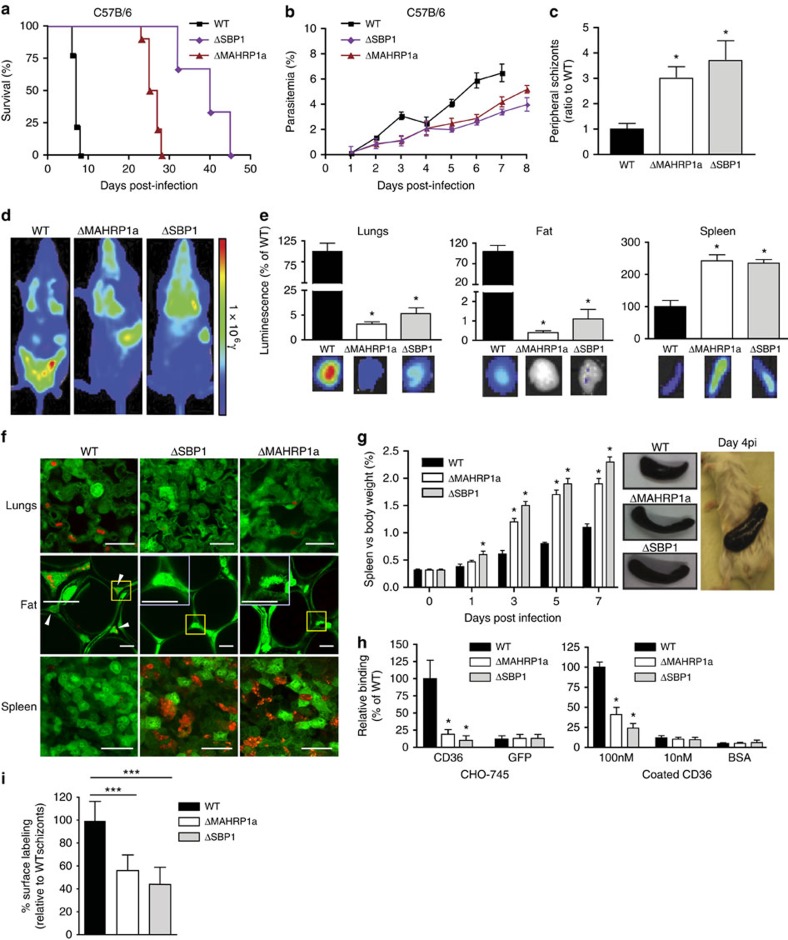
Gene-deletion mutants PbΔ*mahrp1a*and PbΔ*sbp1* show reduced schizont sequestration and virulence. (**a**) Survival of C57B/6 mice infected with mutant and wt parasites (*n*=9 mice per group, *P<*0.001, logrank test from Kaplan–Meier survival curve). (**b**) The course of parasitemia (mean±s.d.) in C57B/6 mice (*n*=15 mice per group) infected with 10^5^ mutant or wt parasites. Significant differences in parasitemia between wt and both mutants at day 3, 6, 7 (*P<*0.001) and day 5 (*P=*0.005) (two-tailed unpaired *t*-test). (**c**) Significant increase in number of mutant schizonts in the peripheral blood circulation compared with wt. Schizonts were quantified in Giemsa-stained smears of tail blood obtained 22 h after establishing synchronised infections (ratio of the number of mutant to wt schizonts, *n*=12 mice per group, *P=*0.01; two-tailed unpaired *t*-test). (**d**) Representative distribution of sequestered schizonts in mice with synchronised infections 22 h post infection of mutant and wt parasites that express luciferase as shown by measuring luciferase activity (photons per s per cm^2^; shown as radiance, *y*; *n*=15 mice per group). (**e**) Quantification of parasite loads in isolated organs after perfusion 22 h after synchronised infections with mutant and wt parasites expressing luciferase. Parasite loads were determined by measuring luciferase activity (photons per s per cm^2^) and expressed relative to wt (*n*=15 mice per group). (**f**) Representative intravital images (spleen, fat) and *ex vivo* (lungs) confocal images of tissues from UBC-GFP mice 22 h after establishing synchronized infections of mutant and wt parasites that express mCherry. Insets in the images of fat tissue are enlargements of the yellow boxes. Arrowheads indicate parasites. Size bars, 10μm. (**g**) Spleens of mutant-infected mice are significantly larger (spleen-to-body weight ratio) than spleens of wt-infected mice (*n*=9 mice per group, *P<0.0001; unpaired Student's *t*- test; infections were done with 10^6^ parasites per mouse). Images of representative spleens at day 4 after infection are shown to the right. (**h**) *In vitro* binding of schizonts of mutant and wt parasites to CD36-GFP-expressing CHO-745 cells (left) and to CD36 immobilized on plates (right). Binding is expressed as % of wt (*n*=6, *P<0.0001; two-tailed unpaired Student's *t*-test). (**i**) Quantitative FACS analysis of surface reactivity of RBCs infected with wt and gene-deletion mutant schizonts cultured *ex vivo* to mouse hyperimmune serum. Both deletion mutant schizonts exhibit significantly reduced levels of surface labelling with *P. berghei* sera compared to wt parasites. The geometric mean fluorescence intensity (MFI) relative to wt is shown (%). (***P, 0.001, unpaired *t*-test).

Next, we generated gene-deletion *P. berghei* mutants that lack the genes encoding PbMAHRP1a and PbSBP1 (PbΔ*mahrp1a* and PbΔ*sbp1*) (Supplementary Fig. 5). While no phenotypic differences were observed between the gene-deletion mutants and wild-type (wt) parasites during development in the mosquito (Supplementary Fig. 6) or in the liver (Supplementary Fig. 7), blood stages of the mutants exhibited an attenuated phenotype in mice. While wt-infected C57B/6 mice died 7–9 days post infection as a result of complications typical for experimental cerebral malaria, PbΔ*mahrp1a*- and PbΔ*sbp1*-infected mice did not develop experimental cerebral malaria but died from hyperparasitemia 25–28 and 32–45 days post infection, respectively (Fig. 3a). A similar attenuation was also observed in infections in Balb/c mice (Supplementary Fig. 8a). The parasitemia in the PbΔ*mahrp1a*- and PbΔ*sbp1*-infected mice was comparable to that of wt during the initial 2 days of infection, but was reduced compared with wt thereafter (Fig. 3b). Both mutants were predominantly found in reticulocytes throughout the course of infection, whereas virulent wt parasites were found in erythrocytes in the later course of infection (Supplementary Fig. 8b). In addition, mice infected with the PbΔ*mahrp1a* and PbΔ*sbp1* mutants showed a 3 and 3.6-fold increase in schizonts in the peripheral blood circulation compared to wt infections, respectively (Fig. 3c), indicating reduced sequestration of RBCs infected with mutant schizonts.

To quantify sequestration of iRBCs *in vivo*, we next generated Pb*mahrp1a* and Pb*sbp1* gene-deletion mutants in *P. berghei* parasites expressing the reporter protein firefly luciferase (PbΔ*mahrp1a*-mCherry_Hsp70_Luc_ef1α_ and PbΔ*sbp1*-mCherry_Hsp70_Luc_ef1α_). Measuring of luminescence signals in synchronous blood stage infections with fully virulent wt parasites at 22 h post infection (when parasites are in the schizont stage) showed a high accumulation of schizont-infected RBCs in the lungs and adipose tissue but low accumulation in the spleen (Fig. 3d), as previously described for *P. berghei* schizonts^8^. In contrast, PbΔ*mahrp1a* and PbΔ*sbp1* parasites showed strongly reduced accumulation in the adipose tissue and lungs compared with wt (Fig. 3d), which is typical for a loss of CD36-mediated sequestration^8^. Conversely, significantly increased accumulation of parasites was detected in the spleens of mice infected with PbΔ*mahrp1a* and PbΔ*sbp1* mutants (Fig. 3d). Higher accumulation in the spleen of non-sequestered schizonts has been attributed to increased removal of non-sequestering schizonts by the spleen^8^. Almost complete absence of mutant parasites from the adipose tissue and lungs, and accumulation in the spleen, was also evident after quantification of parasite levels in perfused organs removed from the mice (Fig. 3e). This phenotype was confirmed by intravital and *ex vivo* imaging of organs with reporter mice-expressing GFP ubiquitously in all tissues and infected with mutant and wt parasites expressing the reporter protein mCherry (Pb-mCherry_Hsp70_, PbΔ*mahrp1a*-mCherry_Hsp70_ and PbΔ*sbp1*-mCherry_Hsp70_) (Fig. 3f; Supplementary Movies 3 and 4).

The spleen plays a key role in clearance of iRBCs in both human and rodent *Plasmodium* infections. It is known to undergo major remodelling, including significant enlargement which has been associated with compromised splenic functions^33^. *P. falciparum* and *P. berghei* parasites are believed to sequester in the peripheral vasculature to avoid elimination in the spleen^3^,^8^. In infections with the PbΔ*mahrp1a* and PbΔ*sbp1* parasites, we observed a strong increase in the size of spleens compared with spleens of wt-infected control mice (Fig. 3g) and intravital imaging showed higher accumulation and passage in the spleen of PbΔ*mahrp1a*-mCherry_Hsp70_ and PbΔ*sbp1*-mCherry_Hsp70_ schizonts compared with control schizonts (Supplementary Fig. 8c). Taken together, the intravital and luminescence data are consistent with decreased sequestration of the mutant parasites in adipose tissue and lungs and increased accumulation and passage in the spleen.

### *In vitro* binding to CD36 depends on PbSBP1 and PbMAHRP1a

The *in vivo* phenotypes of the PbΔ*mahrp1a* and PbΔ*sbp1* mutants indicated reduced sequestration and raised the possibility that the cytoadherence properties of RBCs infected with these parasites were altered. *P. berghei* iRBC containing schizonts were previously shown to bind to CD36 (ref. 9), which is also a major receptor for PfEMP1-mediated cytoadherence in *P. falciparum*^34^,^35^. We therefore carried out *in vitro* binding assays with schizont-infected RBCs. Binding to mouse CD36 was significantly lower in PbΔ*mahrp1a* and PbΔ*sbp1* schizonts compared with wt schizonts with both plate-coated CD36 or CD36 expressed on CHO-745 cells (Fig. 3h). As this mirrors the *in vitro* cytoadherence phenotype observed with the corresponding mutants in *P. falciparum*^17^,^18^,^20^, this suggests that despite the absence of PfEMP1 in *P. berghei*, the machinery mediating surface display of an equivalent parasite ligand is conserved. Loss of ligand surface expression resulting in impaired sequestration may be one reason for the reduced growth and virulence of the mutants *in vivo*.

Loss of PfEMP1 expression in *P. falciparum* parasites results in a reduced reactivity of iRBC to hyperimmune serum of infected patients as measured by surface binding of the serum by FACS^36^. To test whether the reduced adhesion to CD36 in the mutant *P. berghei* parasites was also accompanied by a reduced display of factors expressed on the iRBCs, we carried out similar FACS experiments with hyperimmune sera raised in chronically infected mice. Surface reactivity of hyperimmune sera in PbΔ*mahrp1a* and PbΔ*sbp1* schizont-iRBCs was significantly reduced when compared with wt to 60% and 45%, respectively (Fig. 3i). This supports the idea that MAHRP1a and SBP1 are indeed involved in the transport of certain parasite proteins to the iRBC surface and that this is the reason for the reduced binding to CD36.

### PfSBP1 and PfMAHRP1a complement *P. berghei* orthologues

**Figure 4:**
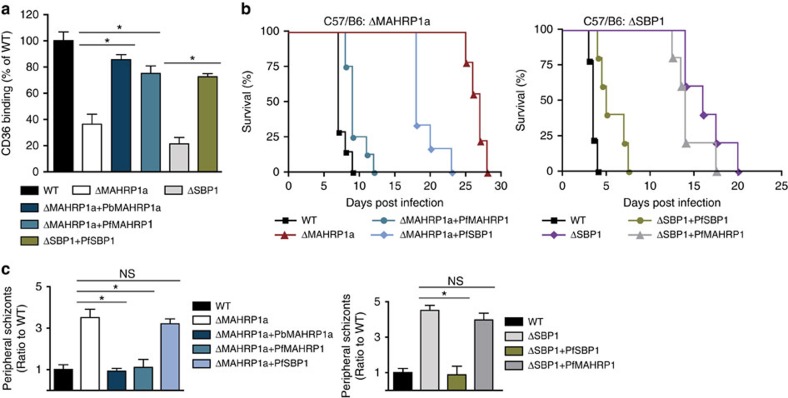
Heterologous complementation of the mutants PbΔ*mahrp1a* and PbΔ*sbp1* with Pf*mahrp1* and Pf*sbp1* rescues binding of iRBCs to CD36 and virulence phenotype. (**a**) The matched complemented mutants showed significantly increased binding to purified CD36 compared with the gene-deletion mutants (binding indicated as % of wt; *P<0.0001, one-way ANOVA, Tukeys *post hoc* test.; *n*=3). (**b**) Survival of C57B/6 mice infected with matched complemented mutants was significantly increased in comparison with the mutants and the mismatched complemented mutants (*n*=9 mice per group, *P<*0.001, logrank test from Kaplan–Meier survival curve). (**c**) Significant decrease in number of schizonts in the peripheral blood circulation in matched complemented mutants compared with the gene-deletion mutants and to mismatched complemented mutants. Schizonts were quantified in Giemsa-stained smears of tail blood obtained 22 h after establishing synchronised infections (*n*=9 mice per group; *P<0.0001, one-way ANOVA, Tukeys *post hoc* test).

To explore further whether the *P. berghei* and *P. falciparum* MAHRP1 and SBP1 have conserved functions, we generated heterologous complementations of the *P. berghei* gene-deletion mutants with the corresponding *P. falciparum* molecules (PbΔ*mahrp1a*+Pf*mahrp1* and PbΔ*sbp1+*Pf*sbp1*; Supplementary Fig. 9). These heterologous complemented mutants were analysed in parallel to a homologous complementation of the PbΔ*mahrp1a* mutant with Pb*mahrp1a* (PbΔ*mahrp1a*+Pb*mahrp1a*). Complementation of PbΔ*sbp1* with Pb*sbp1* was not done, as proper cloning of the corresponding plasmid in *E. coli* was not possible so far (likely due to the >1,000 bp of repeats in this gene). Importantly, both *P. falciparum* molecules, PbSBP1 and PbMAHRP1, restored the *in vitro* binding of iRBC to CD36 of the corresponding mutants to >70% of wt (Fig. 4a). This heterologous complementation provides clear evidence that the bioinformatically identified molecules PbSBP1 and PbMAHRP1a are indeed true functional orthologues of PfSBP1 and PfMAHRP1a, despite their relatively low conservation.

To confirm successful heterologous complementation of the mutants *in vivo*, we infected mice with the corresponding parasite strains (Pf*mahrp1* in PbΔ*mahrp1a* and Pf*sbp1* in PbΔ*sbp1*). The *P. falciparum* molecules restored both the growth phenotype (Supplementary Fig. 10) and the virulence phenotype (Fig. 4b) of the *P. berghei* mutants. The restoration of the phenotypes was specific, as in mismatched complementations (Pf*sbp1* in PbΔ*mahrp1a* and Pf*mahrp1* in PbΔ*sbp1)* that were analysed in parallel, the phenotypes remained similar to the gene-deletion mutants (Fig. 4b; Supplementary Fig. 10). Importantly, the level of schizonts in the peripheral blood circulation in the complemented mutants was comparable to wt parasites and to that of the homologous complementation of the PbΔ*mahrp1a* mutant with Pb*mahrp1a* (Fig. 4c). In agreement with the *in vitro* binding data, this finding suggested restoration of sequestration of the complemented mutants. Conversely, the mismatched heterologous complementations did not lead to reduced schizont levels (Fig. 4c).

**Figure 5:**
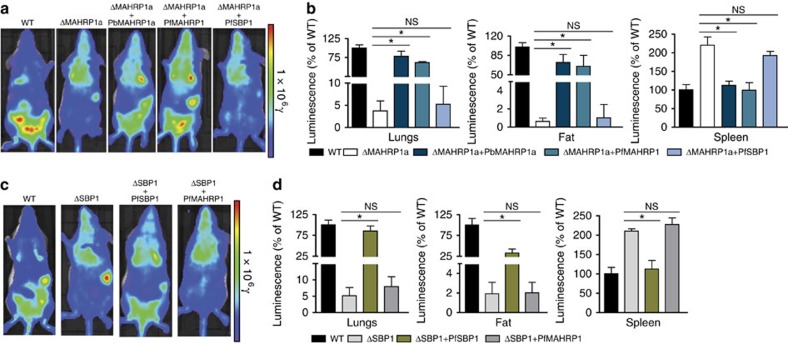
Heterologous complementation of the mutants PbΔ*mahrp1a* and PbΔ*sbp1* with Pf*mahrp1* and Pf*sbp1* rescues the schizont sequestration *in vivo*. Representative distribution of schizonts in mice infected with the matched and mismatched complementation of the PbΔ*mahrp1a* (**a**) and PbΔ*sbp1* (**c**) parasites compared to wt and uncomplemented parasites (photons per s per cm^2^; shown as radiance, *y*). (**b**,**d**) quantification of parasite loads in isolated organs after perfusion (relative luminescence intensity (photons per s per cm^2^) compared to wt-infected mice) obtained 22 h after establishing synchronised infections with luciferase-expressing wt, mutant and complemented parasites (*n*=9 mice per group; *P<0.0001, one-way ANOVA, Tukeys *post hoc* test).

Next, we generated complemented mutants that express luciferase and measured the distribution of schizonts in organs of infected mice by bioluminescence to quantitatively determine their restored sequestration. Complementation of the PbΔ*mahrp1a* mutant with Pb*mahrp1a* restored the schizont sequestration phenotype, with accumulation of schizonts in the lungs and adipose tissue, and reduced accumulation in the spleen (Fig. 5a). The *in vivo* sequestration phenotypes were also restored in the gene-deletion mutants that were complemented with the matched *P. falciparum* molecules (PbΔ*mahrp1a*-mCherry_Hsp70_Luc_ef1α_+Pf*mahrp1*-mCherry_ef1α_ and PbΔ*sbp1*-mCherry_Hsp70_Luc_ef1α_+Pf*sbp1*), whereas no restoration was observed with the mismatched complementations (PbΔ*mahrp1a*-mCherry_Hsp70_Luc_ef1α_+Pf*sbp1* and PbΔ*sbp1*-mCherry_Hsp70_Luc_ef1α_+Pf*mahrp1*) (Fig. 5a,c). Measurement of schizont accumulation in isolated organs after perfusion revealed some interesting subtle differences. Complementation with either the Pb*mahrp1a* or Pf*mahrp1* of PbΔ*mahrp1a* led to restored sequestration in all organs, similar to controls (Fig. 5b). While the heterologous complementation of the PbΔ*sbp1* mutants with Pf*sbp1* (PbΔ*sbp1*-mCherry_Hsp70_Luc_ef1α_+Pf*sbp1*) similarly restored the levels of schizonts sequestering in the lungs and accumulating in the spleen, it showed only 25% restoration of sequestration in adipose tissue at 22 h.p.i. (Fig. 5d). The unmatched complementations showed sequestration levels in the organs that were similar to the gene-deletion mutants, again confirming the specificity of the effect. Taken together these data support an evolutionarily conserved and specific function of the SBP1 and MAHRP1 orthologues that is a prerequisite for sequestration and virulence of iRBCs.

## Discussion

Although iRBC of both *P. berghei* and *P. falciparum* sequester and use similar host cell receptors for adherence to the microvascular endothelium, the similarities of the tissue sequestration-induced virulence between humans and rodents have been debated^6^. We here establish for the first time that components of the trafficking machinery specific for the transport of the ligands exposed on the iRBC that bind to host cell receptors are evolutionarily conserved among malaria parasites, although the ligands themselves are clearly different between human and rodent *Plasmodium* parasites. Our results in the rodent model, in particular the heterologous complementation, further demonstrate that SBP1 and MAHRP1 play an important role in both, organ sequestration and in virulence, results that are difficult to obtain in humans.

So far, the ligand(s) binding to host cell receptors such as CD36 (refs 8, 9) have not been identified in *P. berghei*, despite the identification of proteins encoded by multigene families that are transported to the surface of *P. berghei* iRBCs, specifically members of the rodent-specific Fam-a family^37^. An exported protein, the SMAC, has been implicated in CD36-mediated *P. berghei* iRBC sequestration^9^. However, this protein, encoded by a single copy gene, is not transported to the iRBC surface membrane and orthologs are lacking in human malaria parasites^8^. Further research is required to identify the rodent malaria ligand which will greatly enhance studies on the relationship between iRBC sequestration and virulence in rodent malaria models. The mutants generated in this study lacking expression of SBP1 and MAHRP1a are useful tools to identify such ligands, for example by larger scale screening assays to identify proteins that are absent on the surface of iRBC of these schizonts, for example members of the Fam-a multigene family^37^,^38^.

PbSBP1 and PbMAHRP1a co-localize with the protein IBIS which is a rodent *Plasmodium*-specific protein^39^ that is located in previously described Maurer's clefts-like structures in *P. berghei* iRBC^28^. Our data indicate that these structures in *P. berghei* iRBC indeed serve similar function as the Maurer's clefts in *P. falciparum*. Strong support for this are our observations that *P. falciparum* SBP1 and MAHRP1 were able to complement the functions of PbSBP1 and PbMAHRP1, both with respect to the sequestration and virulence phenotypes. The complemented gene-deletion mutants showed restoration of nearly all phenotypes close to wt, except for the adipose tissue sequestration in the complementation of the SBP1 mutant which was only partly restored. It is possible that complementation results in a lower density of the parasite ligand on the surface compared with wt parasites, which might lead to differences in the sequestration phenotype between tissues with different receptor densities. However, only when the *P. berghei* ligand has been identified, questions related to ligand expression on iRBC and its relationship with sequestration in different organs can be addressed.

The absence of SBP1 and MAHRP1 expression clearly affected the growth rate of blood stages in the mouse. A reduced growth rate had also been observed for a *P. berghei* mutant lacking expression of SMAC that showed a reduced sequestration of iRBC^8^. For this mutant it was shown that the decreased multiplication of blood stages was due to the reduced sequestration phenotype and thereby an increased removal of schizonts from the blood circulation by the spleen. It is therefore likely that the reduced CD36 binding and the ensuing lack of sequestration of the SBP1 and MAHRP1-deficient mutants is at least in part responsible for the reduced growth rate. The important role of the spleen is also clear from the increased accumulation of the gene-deletion parasites in this organ and the strongly enlarged spleens in mice infected with these mutants. This enlargement indicates a marked restructuring of this organ, which may also affect clearance of iRBC as has been previously suggested^40^,^41^.

It is interesting to note that the mutants showed a more restricted preference for growing in reticulocytes compared with wt parasites. Although *P. berghei* ANKA parasites have a strong preference for invading and growing inside reticulocytes^38^, they can invade erythrocytes at higher parasitemias when reticulocytes become a limiting factor^12^,^42^. In the *sbp1* and *mahrp1a* gene-deletion mutants most parasites were found in reticulocytes, even at higher parasitemias. Although the stricter preference of invading reticulocytes may affect the growth rate later in infection, it does not account for reduced sequestration of the *Pbsbp*1 and P*bmahrp*1a gene-deletion mutants since CD36-mediated sequestration of schizonts is independent of their presence in reticulocytes or erythrocytes^9^,^12^.

Our studies highlight unexpected similarities in the molecular machinery underlying parasite sequestration of divergent *Plasmodium* species. We provide the first evidence for the presence of proteins in Maurer's clefts-like structures that play a role in sequestration of rodent malaria and that are orthologues of proteins involved in the transport of the major sequestration and virulence protein PfEMP1 of the human parasite *P. falciparum*. The conservation of this machinery indicates that the rodent malarias can be powerful models to study sequestration and virulence *in vivo* with relevance for human disease and that the creation of falciparumized *P. berghei* expressing *P. falciparum* virulence factors may be feasible. Such transgenic *P. berghei* parasites would be invaluable tools for testing inhibitors that block *P. falciparum* sequestration.

## Methods

### Identification of SBP1 and MAHRP1a in other *Plasmodia*

PfSBP1 and PfMAHRP1 protein sequences were used in BLAST searches in Plasmodb (v5.5) to identify the orthologues in other *Plasmodium* species. Initially the now annotated orthologues in *Plasmodium vivax* and *Plasmodium knowlesi* were identified and used as further sequences to conduct BLAST searches in PlasmoDB. This revealed the rodent *Plasmodium* homologues.

To confirm the sequence initially identified by BLAST, we used a HMM-based approach to identify orthologues of PfSBP1 and PfMAHRP1: low complexity amino-acid regions of PfSBP1 and PfMAHRP1 were removed with the SEG algorithm^43^, using the following parameter: window=12; lowcut=2.3 and highcut=2.5. Subsequently, a jackhmmer search^44^ was conducted against the annotated protein sets of *P. falciparum* (3D7), *P. vivax* (Sal-1), *P. knowlesi* (H), *Plasmodium cynomolgi* (B), *Plasmodium chabaudi* (AS), *Plasmodium yoelii* (17XNL) and *P. berghei* (ANKA), as deposited in PlasmoDB v11.1 (ref. 45), using an *e*-value inclusion threshold of 0.001. Orthologues were aligned with MAFFT v7 (ref. 46) using the E-INS-i routine^47^.

For phylogenetic analyses, the best fitting amino-acid substitution models were selected with ProtTest v2.4 (ref. 48) using the Akaike information criterion and Bayesian analyses were conducted with MrBayes v3.2 using the following parameters: JTT+G and JTT+I+G models were used for SBP1 and MAHRP, respectively; analyses were run for 5,000,000 generations; every 1,000th generation was sampled and the final 37,500 samples were used for tree reconstructions.

### Animals

The mouse lines used were Balb/c mice (Harlan laboratories, Netherlands, and University of Bern Tierstall, Switzerland), C57B/6 mice (Harlan laboratories, Netherlands); and transgenic UBC-GFP C57B/6 mice (Institute of Cell Biology (IZB), University of Bern, Switzerland). All studies were done in female 5–8-week-old mice. Mice used for bioluminescence experiments were housed under specific pathogen-free conditions at the Theodor Kocher Institute (TKI) of the University of Bern, Switzerland. All other mice were housed at IZB (University of Bern, Switzerland). Mice were maintained under a 12 h light and 12 h dark cycle. All experiments were performed with the approval of the Animal Research Ethics Committee of the Canton Bern, Switzerland (Permits 81/11 and 105/10), and the University of Bern Animal Care and Use Committee, Switzerland. All measurements performed for either bioluminescence or intravital microscopy, were performed under isofluorane or xylazine/ketamine anaesthesia, and all efforts were made to minimize suffering.

### *P. berghei* ANKA reference lines

Three *P. berghei* ANKA parasite lines were used as reference: wt *P. berghei ANKA*; line 1804 mCherry_Hsp70_ (from the *PbANKA* GIMO motherline 1596cl1, Leiden)^49^; and line 1868 mCherry _Hsp70_Luc_ef1α_ (from the *PbANKA* 1596cl1 GIMO motherline, Leiden)^50^.

### Plasmid constructs

To express a C-terminal GFP fusion of PbSBP1 in *P. berghei*, the full-length coding region of Pb*sbp1* was PCR amplified with primers PbSBP1-bam-fw and PbSBP1-bam-rv (all the primers used in this work are described in Supplementary Table 1) and cloned into *Bam*HI digested pL0017; this plasmid had been obtained through the Malaria Research and Reference Reagent Resource Centre (MR4) deposited by Andy Waters (MR4 no., MRa-786). For the expression of PbSBP1-GFP in *P. falciparum*, its coding region was PCR amplified using primers PbSBP1-KpnI-fw and PbSBP1-AvrII-rv and cloned *Kpn*I/*Avr*II into pARL1a^51^. To test for exporting regions in *P. berghei* PNEPs, the sequence encoding the first 20 amino acids of PbMAHRP1a-GFP and PbSBP1 were fused to the mTRAP reporter^31^. For this the primer KpnI-PbMAHRP1a1-20-mTRAP_F and KpnI-PbSBP1-1-20-mTRAP_F, were each used with primer mTRAP rev (*Avr*II)^31^ to obtain the respective inserts which were cloned *Kpn*I/*Avr*II into pARL1a-GFP.

To generate the gene-deletion constructs, 1,129 bp of the PbSBP1 5′UTR (including the first 41 bp of the gene) and 1,000 bp immediately downstream of the stop codon were PCR amplified with primers PbSBP1-5utre-kpnfw2 and PbSBP1-5′-utr-aparv2 as well as PbSBP1-3′utr-ecorIfw and PbSBP1-3′utr-notrv, respectively and cloned *Kpn*I/*Apa*I and *Eco*R1/*Not*I into pOBconGFP^52^, a derivate of pOB90 kindly donated by Oliver Billker. This resulted in plasmid pOBconGFP-PbΔSBP1. To generate the *mahrp1*a gene-deletion plasmid, 1,017 bp immediately upstream of the start ATG were PCR amplified with the primers PbMAHRP1a-5′utr-kpnfw and PbMAHRP1a-5′utr-aparv and 1,007 bp immediately downstream of stop codon were PCR amplified with primers PbMAHRP1a-3′utr-ecorIfw and PbMAHRP1a-3′utr-notrv and cloned *Kpn*I/*Apa*I and *Eco*R1/*Not*I into pOBconGFP to obtain pOBconGFP-PbΔMAHRP1a.

To generate the complementation constructs, *tgdhfr/ts* in pL0017mCherry^53^ was replaced with *hdhfr/ts* (PCR amplified with primers hDHFR-F-AgeI and hDHF-R-NheI) using *Age*I and *Nhe*I to obtain pL17mCherry-hDHFR. Pb*mahrp1a* was then PCR amplified with primers PbMAHRP1a_fw_BamHI and PbMAHRP1a_rev_AvrII_part 2A_1 for 5 cycles followed by a second full PCR where the reverse primer was exchanged with PbMAHRP1a_rev_ClaI_part 2A_part mCherry_MscI_2. The resulting product contained the full *mahrp1a* gene followed by an *Avr*II site, the 2A skip peptide^54^,^55^, a *Cla*I site, 28 bp of *mcherry* to span the sequence to the *mcherry* internal *Mscl*II site and a *Mscl*II site for cloning the product into pL17mCherry-hDHFR. The PCR product was cloned *Bam*HI/*MscI*II to obtain pL17-Pb*mahrp1a*-2A-mCherry^hDHFR^ and renamed pL17-PbMAHRP1a-AB. To generate the heterologous add backs, this construct was digested with *Bam*HI and *Avr*II to replace Pb*mahrp1a* with the genes encoding PfSBP1 and PfMAHRP1 (PCR amplified with the primers PfMAHRP1_fw_BamHI and PfMAHRP1_rev_AvrII, as well as PfSBP1_fw_BamHI and PfSBP1_rev_AvrII) to obtain pL17-PfMAHRP1a-AB and pL17-PfSBP1-AB.

To obtain the plasmids for recombinant expression of parts of PbSBP1 and PbMAHRP1a GST fusion proteins the DNA sequences encoding amino acids 494–600 (PbSBP1mid) and 601–723 (PbSBP1-C) of PbSBP1 as well as amino acids 127–179 of PbMAHRP1a were cloned *Bam*HI and *Xho*I into pGEX-6-P2 (GE Healthcare). The corresponding PCR products were generated using primers PbSBP1_mid_fw_BamHI and PbSBP1_mid_rev_XhoI (PbSBP1mid), PbSBP1_C_fw_BamHI and PbSBP1_C_rev_XhoI (PbSBP1-C), and PbMAHRP1a_C_fw_BamHI and PbMAHRP1a_C_rev_XhoI (PbMAHRP1a). Inserts of all plasmids were sequenced to verify that there were no undesired mutations.

### Recombinant protein expression and production of antisera

PbMAHRP1a and PbSBP1 GST fusion proteins were expressed in *E. coli* BL21 cells and purified using glutathione-Sepharose (GE Healthcare) according to standard procedures. Antisera were raised commercially (Eurogentec) in rabbits. The sera were purified using the Nab Protein A Plus Spin Kit (Pierce) according to the manufacturer's instructions.

### Live cell imaging and immunofluorescence assays

Immunofluorescence assays (IFAs) were carried out by one of two methods. Acetone fixation of cells dried on multiwell slides were done essentially as described^56^. Briefly, blood of infected mice were washed in PBS, applied to 10-well glass slides and allowed to air dry. The slides were placed into 100% acetone for 20 min. As an alternate new method, cells were first coated onto the multiwell slide using ConcanavalinA as described^56^, followed by aspiration of all excess liquid on the well followed by immediate submersion in 100% acetone before the slide can dry. This method was required to detect PbMAHRP1a. Cells fixed with either method were re-hydrated by adding 1 × phosphate-buffered saline (PBS) to the wells followed by one wash in 1 × PBS. The cells were then blocked in 3% BSA in 1 × PBS and primary sera were applied for 1 h in blocking solution. The wells were then washed 5 × in 1 × PBS, the secondary antibody applied for 1 h in blocking solution containing 1 μg ml^−1^ 4′,6′-diamidine-2′-phenylindole dihydrochloride (DAPI) (Roche). The primary antibodies were diluted 1/10,000 (rabbit anti-PbSBP1-mid), 1/5,000 (rabbit anti-PbSBP1-C) and 1/1,500 (rabbit anti-PbMAHRP1a), and were all newly raised (see ‘Recombinant protein expression and production of antisera'). Rrabbit anti-REX1^30^ was diluted 1/5,000 and the mouse monoclonal anti-GFP (Roche) was diluted 1/500. Secondary antibodies used were goat anti-mouse Alexa Fluor 488, donkey anti-rabbit Alexa Fluor 594 and goat anti-rabbit Alexa Fluor 647 (Life Technologies). The slides were mounted using DAKO antifade and imaged with an Axioimager M1 equipped with a 100 × /1.4 immersion lens and a Hamamatsu Orca C4742-95 camera. Zeiss Axiovison software was used to capture images. *P. falciparum* parasites expressing GFP-fusion proteins were viewed in RPMI medium after staining the nuclei with 1 μg ml^−1^ DAPI (Roche) for 10 min (ref. 57). Images were processed in Corel Photo-Paint X5. Quantification of export was done as described^31^ by counting of cells showing (i) only export, (ii) a mix of exported and non-exported GFP signal and (iii) no export. This was done blinded on three occasions by two researchers counting at least 50 cells per event per person^31^. For three-dimensional reconstructions z-stacks were acquired using an Olympus FV1000 confocal microscope and images processed with Imaris (Bitplane). For time-lapse imaging, cells were imaged with a Zeiss Observer. Z1 confocal line scanning inverted microscope integrated into an LSM5 Live imaging setup. Images were acquired using a Zeiss 63x Plan-Apochromat 1.4 Oil objective and the Zeiss Efficient Navigation 2009 software. After acquisition, contrast and brightness levels were optimized using Image J software.

### Intravital imaging and parasite passage

Five to 6-week-old female UBC-GFP C57B/6 mice were used for all intravital imaging. Mice were infected with either Pb-mCherry_Hsp70_, PbΔ*mahrp1a*-mCherry_Hsp70_ or PbΔ*sbp1*-mCherry_Hsp70_ parasites. After reaching 5–6% peripheral parasitemia, blood was isolated by cardiac puncture, and an *in vitro* schizont culture set up as previously published^58^. Naive mice were infected with 10^6^ schizonts, and 21–24 h later, intravital microscopy was performed. A mixture of anaesthetics comprising 125 mg kg^−1^ ketamine and 12.5 mg kg^−1^ xylazine, was prepared and diluted in PBS (1:2:5). Mice were injected with 100 μl per 20 g of the anaesthetic at 19 h post infection. The surgical procedure involved a small incision to expose the spleen. The exposed spleen was glued to a microscope cover glass (VWR, 24 × 50 mm, No.1), and imaged with a Zeiss LSM5 confocal microscope, using a 63 × oil immersion objective. Animal body temperature was maintained at 37 °C using a temperature-controlled incubation chamber. Simultaneous imaging was performed with the HeNe 488 and HeNe 594 lasers to acquire GFP and mCherry images from the host and parasite respectively, in a 512 × 512 field of view (speed 4, zoom 1.5, Pinhole 1.0). Between 50 and 100 different fields of view were selected in the mouse spleen, and images acquired every 500 ms for 5 min. Experiments were done in triplicate mice for each mutant and wt parasite line. Line scans were positioned at various depths of the vessel, and video-rate imaging was used to record blood and parasite flow in mutant and wt *P. berghei*-infected mice. We generated algorithms to calculate blood flow through various vessel subsets in each movie produced. Vessels away from bifurcations, and with high signal-to-noise ratio, were selected for further image processing. During intravital imaging, the adipose tissue, and liver were similarly imaged. These and the lungs, heart, and kidneys, were extracted, embedded in O.C.T. compound (Tissue Tek), and frozen for 20 μm cryosections and fluorescence image analysis that was carried out with a Leica SP8 microscope, 63 × oil immersion lens. Images were analysed by Imaris and Fiji.

### Protein extraction and western blotting

For *P. berghei* parasite protein extracts, blood of infected mice was washed twice in 1 × PBS and infected red blood cells purified on a Percoll gradient^59^ as described for *P. falciparum*^60^. The purified infected RBCs were washed thrice with 1 × PBS, the pellet lysed in Laemmli sample buffer and the proteins separated on 10% SDS PAGE gels followed by transfer to nitrocellulose membranes (Schleicher & Schüll) in a tank blot device (BioRad). The blots were blocked in 5% milk in 1 × PBS and reacted with the following rabbit-derived antibodies: anti-PbSBP1-mid (1:200'000), anti-PbSBP1-C (1:1'000), anti-PbMAHRP1a (1/500), anti-PfSBP1 (1/1,000) and anti-PfMAHRP1 (a kind gift of Hans-Peter Beck) (1/2,500) for 2–4 h hours. After 5 washes in 1 × PBS, goat anti-rabbit horseradish peroxidase-conjugated secondary antibody, diluted 1/2,500 (Dianova), was applied for 1 h, the membrane washed, reacted with ECL (GE Healthcare) and the signal detected using X-ray films. Alternatively, secondary antibodies were anti-rabbit IgG 800CW IRDye or IgG 680LT IRDye (both 1:10'000) and an Odyssey Imaging system (Li-Cor Bioschiences) was used for detection.

### Generation of transgenic PbΔ*sbp1* and PbΔ*mahrp1a* parasites

Transfection and selection of *P. berghei* ANKA mutant parasite lines was performed as previously described^58^. Briefly, the pOBconGFP-PbΔSBP1 and pOBconGFP-PbΔMAHRP1a plasmids, were linearized (using *Not*I and *Kpn*I for pOBconGFP-PbΔSBP1 and *Not*I and *Sap*I, or *Not*I and *Pvu*II for pOBconGFP-PbΔMAHRP1a). Schizonts from each respective reference line were isolated, and transfected with the linearized plasmids using the nonviral Nucleofactor technology. Transfected parasites were injected by intravenous injection into Balb/c mice. PbΔ*mahrp1a* and PbΔ*sbp1* mutant parasites were selected via administration of 70 μg pyrimethamine per ml of drinking water. Following successful selection of transgenic parasites (as determined by fluorescence), mice were bled by intracardiac puncture and screened by integration-PCR for determination of gene-deletion status. Several clonal PbΔ*mahrp1a* and PbΔ*sbp1* parasite lines were generated in two ways, namely, limiting dilution and detached cell injection, as previously described^58^,^61^. In the resulting clonal lines, correct integration of the DNA constructs was determined by PCR using the primers listed in Supplementary Table 1. Green fluorescence of the gene-deletion parasites was confirmed microscopically.

### Complementation of PbΔ*mahrp1a* and PbΔ*sbp1* lines

Two different clones of the PbΔ*mahrp1a* and PbΔ*sbp1* lines in the wt and the Pb1868 (Pb-mCherry_Hsp70_Luc_ef1α_) background were selected, and used for generation of complemented lines. Constructs pL17-PfMAHRP1a-AB and pL17-PfSBP1-AB encoding full-length *P. falciparum* MAHRP1 and SBP1, as well as *P. berghei mahrp1a* (construct pL17-PbMAHRP1a-AB) were used to transfect the PbΔ*mahrp1a* and PbΔ*sbp1* lines. Parasites containing the constructs were selected by administration of WR99210 into mice and cloned, resulting in the transgenic lines PbΔ*sbp1*+Pf*sbp1*, PbΔ*sbp1*+Pf*mahrp1*, PbΔ*mahrp1a*+Pf*mahrp1*, PbΔ*mahrp1a*+Pf*sbp1* and PbΔ*mahrp1a*+Pb*mahrp1a*. Green and red cytoplasmic fluorescence of the add-back parasites was confirmed microscopically.

### Mosquito-stage characterization

Five- to six-week-old female Balb/c mice were pre-infected with blood stage transgenic parasites and maintained under pyrimethamine treatment in the drinking water. Upon first appearance of parasites in the peripheral blood, naive 5–6-week old female Balb/c mice were phenylhydrazine (PH)-treated. Two–three days post PH-treatment, the pre-infected mice were bled by intra-cardiac puncture, and infected blood was passaged into the PH-treated mice. Three days post infection, upon observation of exflagellation in the peripheral blood, the infected mice were used to feed *Anopheles stephensi* female mosquitoes. Ten to eleven days following the blood meal, infected mosquitoes were sorted via fluorescence detected in the midguts. Pools of 30 infected mosquitoes per *P. berghei* line were dissected and the midguts isolated to evaluate oocyst formation, timing and numbers. Oocysts were fixed in 2% PFA for 10 min, and imaged. Quantification of infected midguts from the different clones was performed using a previously described semi-automated method^62^. At days 16–28 post-blood meal, salivary glands were dissected, and sporozoites isolated from 60 mosquitoes pooled into groups of 5 in 2 independent experiments. The tubes containing the collected sporozoites were coded and sporozoite numbers were counted using a Neubauer chamber, and compared among *P. berghei* transgenic and wt lines. Counting was blinded.

### HeLa cell culture and *in vitro* liver-stage infection assays

In parallel to mosquito-stage characterizations, sporozoites from wt and gene-deletion lines were isolated to perform *in vitro* infections of HeLa cells as described below. HeLa cells (a generous gift by Robert Menard, Pasteur Institute, Paris) were maintained in complete MEM containing Earle's Salts Medium, supplemented with 10% heat-inactivated fetal calf serum (FCS), 1% L-Glutamine and 1% penicillin-streptomycin. Cells were maintained at 37 °C in a 5% CO_2_ cell incubator. Four times 10^4^ cells were seeded into 24-well plates for infection assays. Sporozoites of wt and gene-deletion parasites were prepared from dissected salivary glands, incubated in complete MEM media containing Amphotericin B (2.5 μg ml^−1^) (10 × -AT medium), and added to the 24-well plates containing monolayers of HeLa cells. Following infection, cells were incubated with 1 × AT medium at 37 °C and 5% CO_2_ for 12, 24, 36, 48, 56 and 60–65 h. At 12 h post infection, wt and mutant parasites were compared in their HeLa cell invasion capacity. At 24, 36, 48 and 56 h post infection, wt and gene-deletion lines were analysed by fixed immunofluorescence assay for size and PVM morphology. At least 100 infected cells were imaged and further analysed. Cells were fixed with 4% paraformaldehyde, permeabilized with 0.1% Triton X, and stained with anti-GFP or anti-RFP; anti-EXP1 and/or anti-UIS4 antibodies, and DAPI. Parasites from all time points were compared in terms of size and PVM morphology. Finally, detached cell/merosome generation—as a marker of successful liver cycle completion—was quantified at 60–65 h post infection and compared between wt and mutant parasites.

### Determination of parasitemia and survival assays

To assess parasitemia over time and for the survival assays, 6-week old female Balb/c and C57B/6 mice were infected by intravenous (i.v.) injection, with 10^4^–10^6^ schizonts from *in vitro* schizont cultures^58^ of individual clones of wt, PbΔ*mahrp1a* and PbΔ*sbp1* lines in the mCherry_Hsp70_Luc_ef1α_ background. From day 1 post infection, Giemsa-stained blood smears were obtained for analysis of parasitemia and life cycle stage distribution, leukocytes and additional haematological features. Additionally, parasites were collected for bioluminescence *in vitro* assays, and FACS analysis for total parasitemia determination. Parasitemia was followed daily until the mice exhibited symptoms of anaemia or the onset of cerebral malaria, at which point the mice were euthanized. We assessed the gene-deletion mutant or wt status of the parasites with overall mouse survival in a univariate Cox proportional hazards model, and Kaplan–Meier curves. Parasitemia growth and reticulocyte preference were quantified by two independent observers one of whom was blinded to the wt or mutant status of the parasites and the time post infection represented in the Giemsa smears. Experiments were repeated at least 3 times, with 5 mice per parasite line.

### Bioluminescence *in vivo* and organ *ex vivo* imaging

Schizont sequestration was imaged using luciferase-expressing wt, gene-deletion mutant, and complemented parasites, in a bioluminescence *in vivo* imaging system (IVIS 100; Caliper Life Sciences—f/stop, 1; no optical filter) both, *in vivo* (3 min exposure; 10 min post-substrate injection; 8 × binning; 12.5 cm field view) and *ex vivo* (30 s exposure and 3 min post-substrate injection; 8 × binning; 10 cm field view). Sequestration patterns at 22–24 h post invasion in synchronous infections were monitored in Balb/c and C57B/6 mice, and normalised to schizont levels of the wt control. Synchronized infections were established by schizont purification, isolation, and injection as previously described. Mice were anaesthetized using 2.5% isofluorane, and 100 μl of the substrate RediJect were injected intraperitoneally. Following whole-body imaging, mice were euthanized, and perfused with 1 × PBS to remove unbound schizonts. Individual organs including the lungs, heart, liver, adipose tissue and spleen, were removed from infected mice following perfusion. Acquired data were analysed using the programme Living Image 4.4. Experiments were repeated a minimum of three times, with 3–5 mice per parasite line.

### Histology

For histology, UBC-GFP transgenic C57B/6 or wt C57B/6 mice were fixed with 2% PFA by intracardiac perfusion. Adipose tissue, lungs, liver, heart, kidneys and spleens were removed for further processing. Fixed tissues were embedded in paraffin and 20–50 μm sections were stained with H&E or Giemsa, and number of parasites per vessel area was quantified. The organs of at least 3 mice per parasite line were analysed by histology.

### Splenomegaly

To determine splenomegaly scores, the spleens of mice infected with gene-deletion mutant, wt and complemented lines were removed at various time points post infection (uninfected controls, and days 1, 3, 5 and 7 post infection). Before removal of the spleens, mice were weighed. After removal, the weight of the spleen was recorded for percentage calculation (spleen-to-body weight).

### *In vitro* CD36 binding assays

For *in vitro* measurement of the adhesion of mutant and wt *P. berghei* parasites to CD36-coated dishes recombinant mouse CD36/Fc chimera (purchased from R&D) was used. Triplicate plastic dishes were coated with protein A (BioVision) diluted at 20 μg ml^−1^ in PBS (pH 9.0) or PBS-containing 1% (wt/v) bovine serum albumin (BSA) for the control for 1 h at 37 °C in a cell culture incubator. Dishes were washed three times with PBS (pH 7.4) to remove unbound protein, and blocked with PBS+1% BSA at 4 °C overnight. After washing once with PBS, plates were coated with recombinant mouse CD36 (100, 10, 5, 2 and 1 nM) or PBS-containing 1% BSA (cell culture grade) for 3 h at 37 °C in a cell culture incubator. Plates were washed a further three times with PBS, and incubated once more for 30 min with 1% BSA in PBS. In parallel, schizonts from all strains were isolated following 16 h of *ex vivo* maturation as previously described^8^,^58^. The matured schizonts (10^6^) were added to binding medium (RPMI-1640, 25 mM HEPES, 25 μg ml^−1^ gentamycin, 2 mM L-glutamine, 10% FCS) and allowed to bind in individual compartments for 1 h. After 1 h, dishes were placed in an orbital shaker for 1 h, and finally, washed with PBS before measurement of bound parasites by fluorescence microscopy. Each condition was done in triplicate, and each experiment repeated 3–5 times. The differential binding of wt versus gene-deletion mutant parasites was determined based on quantification of bound parasites per mm^2^ by mCherry fluorescence.

For binding assays with CD36 expressed on cells, the following Chinese hamster ovary (CHO) cells were used as per a setup previously described for *P. falciparum*^63^. CHO-K1 (a kind donation by Phillip Odermatt and Daniel Schuemperli), CHO-745 (a kind donation by Beat Trueb) and CHO-745 transfectants expressing CD36, were cultured in DMEM medium supplemented with 10% fetal calf serum, 1% L-glutamine, 1% L-proline and 1% penicillin/streptomycin. CHO-745 cells were transfected with GFP-tagged CD36, using nucleofactor technology. Receptor surface expression in transiently and stably transfected cells was monitored by flow cytometry on a weekly basis. For binding assays, individual CHO cell lines were grown to 70% confluence, and 10^4^ cells seeded in 33 mm polystyrene dishes (*In Vitro* Scientific) 24–36 h before binding assays. For binding, CHO cells were washed three times with pre-warmed binding media (RPMI-1640 plus 0.1% BSA). Schizonts were purified as previously published^8^,^58^, and 10^6^ schizonts overlayed onto the CHO cells and incubated for 1 h at 37 °C. Thereafter, non-binding erythrocytes were removed by transferring the binding dishes to an orbital shaker for 30 min, and then by gently flooding the dishes with warm binding medium and replacing the medium three times. Binding of wt and gene-deletion parasite mutants was quantified under a confocal microscope (Leica SBP8, 100 × objective) or a fluorescence microscope (Leica L550b), by determining the number of schizonts adhering to at least 500 cells. Binding was expressed as number of schizonts bound per 100 cells. The binding experiments were performed by two independent researchers, one of whom was blinded to the mutant or wt status of the parasites.

### Flow cytometry to analyse *P. berghei* surface antigens

FACS-based detection of surface antigens was done similarly to what was previously published^15^. Mice infected with wt, MAHRP1- and SBP1-deletion mutants were exsanguinated by cardiac puncture at day 5 post infection (5–10% parasitemia). Establishment of *in vitro* schizont cultures and isolation of schizonts were carried out as previously described^58^. Schizonts were washed 1 × in RPMI and blocked for 1 h in 1% casein in RPMI (blocking solution). Six million schizonts per condition were incubated for 1 h in serum harvested from *P. berghei* semi-immune mice previously infected with wt parasites, (or non-immune mice), essentially as described previously^64^: C57B/6 mice were infected with wt *P. berghei*, and 5 days post infection treated with a curative dose of chloroquine and sulfadiazine. Following disappearance of parasites from the periphery, the mice were re-infected, and the procedure repeated a total of four times, after which the serum was isolated. The serum was used 1:20 in blocking solution. After three washes with blocking solution, cells were incubated for 1 h in the dark, with goat anti-mouse Cy5 (1:2,000, Dianova), then washed a further three times. Following the last wash, schizonts were resuspended in 500 μl of 1 × PBS, and analysed with a BD LSR II Flow Cytometer (BD Biosciences). Gating was performed using BD FACSDiva Software (BD Biosciences), and analysis performed using FlowJo. All incubations were performed at 20–22 °C. Following gating for erythrocytes and gating for eliminating duplets, infected red blood cells were gated based on their tagging with GFP and/or mCherry. Serum IgG binding for each sample (surface labelling) was expressed as the geometric mean fluorescence intensity (MFI) relative to wt, after subtracting the MFI of uninfected erythrocytes.

### Statistical analyses

All experiments were repeated a minimum of three times. Mouse experiments were repeated with 3–5 mice per group each time. Data are expressed as mean±s.e.m. The main statistical test used was the Student's *t*-test. For experiments involving multiple comparisons, we used multivariate analyses of variance followed by *post hoc* within and between groups hypothesis testing. All analyses were performed using STATA 13. All *P* values less than 0.05 were considered to be statistically significant.

### Data availability

The authors declare that the data supporting the findings of this study are available within the article and its supplementary information files, or from the corresponding authors upon request.

## Additional information

**How to cite this article:** De Niz, M. *et al*. The machinery underlying malaria parasite virulence is conserved between rodent and human malaria parasites. *Nat. Commun.* 7:11659 doi: 10.1038/ncomms11659 (2016).

## Supplementary Material

Supplementary Information Supplementary Figures 1-10, Supplementary Table 1

Supplementary Movie 1 Rotation of 3D-reconstructed *P. berghei* iRBCs labelled with anti-PbSBP1 serum by IFA shows a staining pattern reminiscent of Maurer's clefts in *P. falciparum*.

Supplementary Movie 2 2D-time lapse imaging shows that PbSBP1-GFP labelled structures in *P. berghei* iRBCs are mobile.

Supplementary Movie 3 Intravital microscopy of UBC-GFP mouse spleens infected with control parasites.

Supplementary Movie 4 Intravital microscopy of UBC-GFP mouse spleens infected with PbΔSBP1 mCherryHsp70 parasites.
