# Supplementary material for: The machinery underlying malaria parasite virulence is conserved between rodent and human malaria parasites
Source: Nat Commun. 2016 May 26;7:11659. doi: 10.1038/ncomms11659 (PMC4894950; doi:10.1038/ncomms11659)
Supplement: Supplementary Information — Supplementary Figures 1-10, Supplementary Table 1 [file ncomms11659-s1.pdf]

a

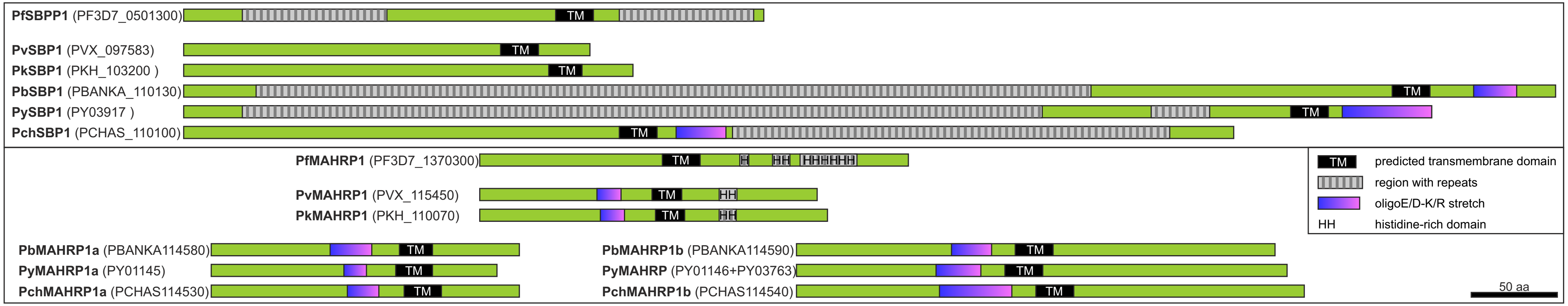

b

	PfaSBP1	PknSBP1	PviSBP1	PcySBP1	PchSBP1	PbeSBP1	PyoSBP1
PfaSBP1	---	51.0	50.0	39.2*	44.4	45.3	49.8
PknSBP1	26.0	---	78.0	62.5*	45.5	40.2	45.8
PviSBP1	24.5	62.9	---	76.2*	52.5	42.8	46.9
PcySBP1	19.2*	49.2*	62.3*	---	47.1	35.3	39.8
PchSBP1	18.1	22.7	28.2	26.0	---	74.1	73.8
PbeSBP1	17.9	21.7	22.5	19.1	63.2	---	75.4
PyoSBP1	19.3	23.2	24.5	21.4	62.8	64.6	---

	PfaM1	PknM1	PviM1	PcyM1	PchM1a	PbeM1a	PyoM1a	PchM1b	PbeM1b	PyoM1b
PfaMAHRP1	---	57.4	60.7	50.0	43.1	39.7	42.3	74.7	72.3	69.0
PknMAHRP1	32.4	---	79.1	69.9	45.3	40.6	47.0	45.3	40.0	37.1
PviMAHRP1	37.3	62.8	---	76.5	50.0	47.5	51.6	49.4	44.5	39.6
PcyMAHRP1	24.3	48.8	62.3	---	54.6	54.5	62.8	44.2	42.8	38.4
PchMAHRP1a	19.7	17.3	22.2	17.6	---	73.4	72.3	46.7	48.5	49.7
PbeMAHRP1a	15.6	14.7	23.0	20.9	57.2	---	84.3	41.3	46.0	43.8
PyoMAHRP1a	16.9	19.7	26.6	27.7	50.0	71.1	---	44.2	46.1	44.2
PchMAHRP1b	29.9	20.6	22.6	20.3	25.4	24.0	25.0	---	75.4	78.1
PbeMAHRP1b	30.1	20.0	18.9	18.1	23.6	24.0	26.3	60.2	---	87.6
PyoMAHRP1b	28.7	17.1	18.9	17.4	23.7	23.4	26.3	60.2	76.7	---

c

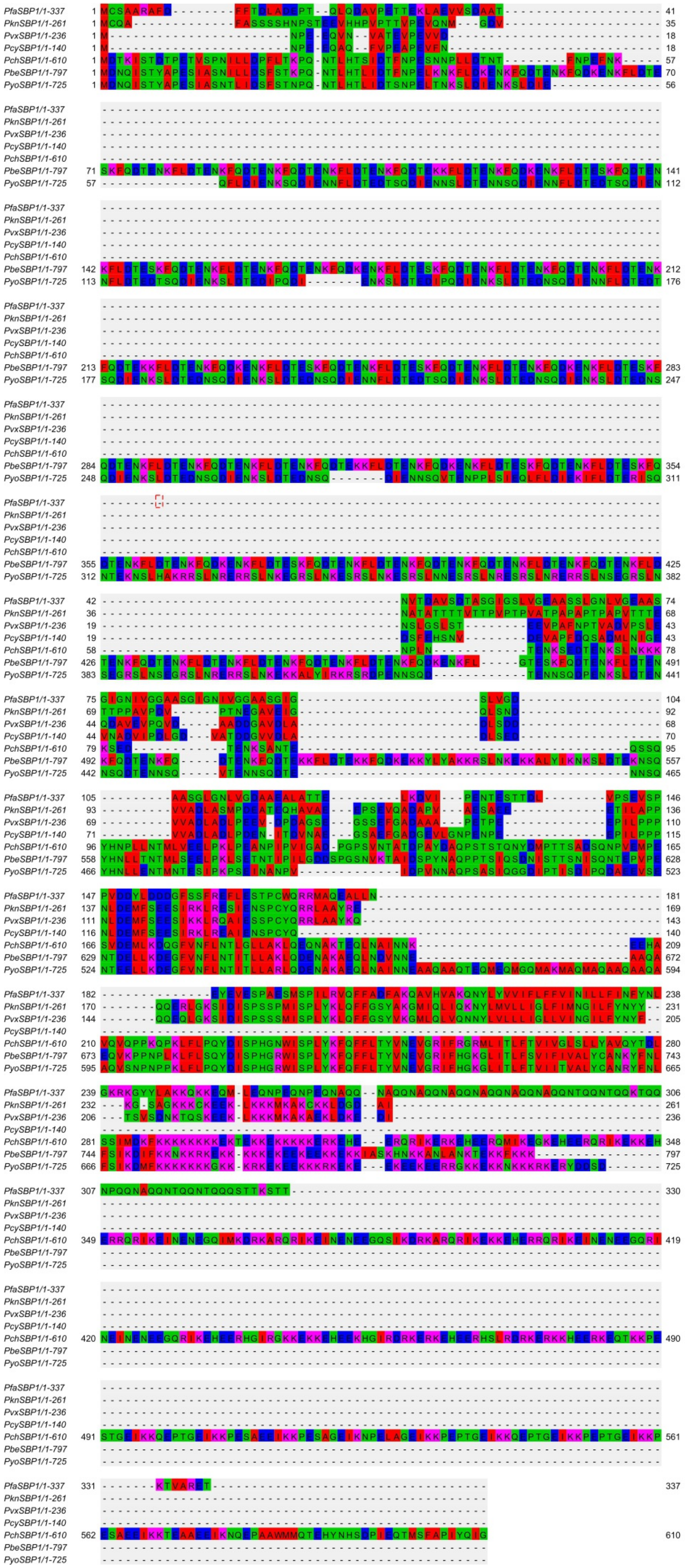

PfaSBP1/1-337
PknSBP1/1-261
PvxSBP1/1-236
PcySBP1/1-140
PchSBP1/1-610
PbeSBP1/1-797
PyoSBP1/1-725

d

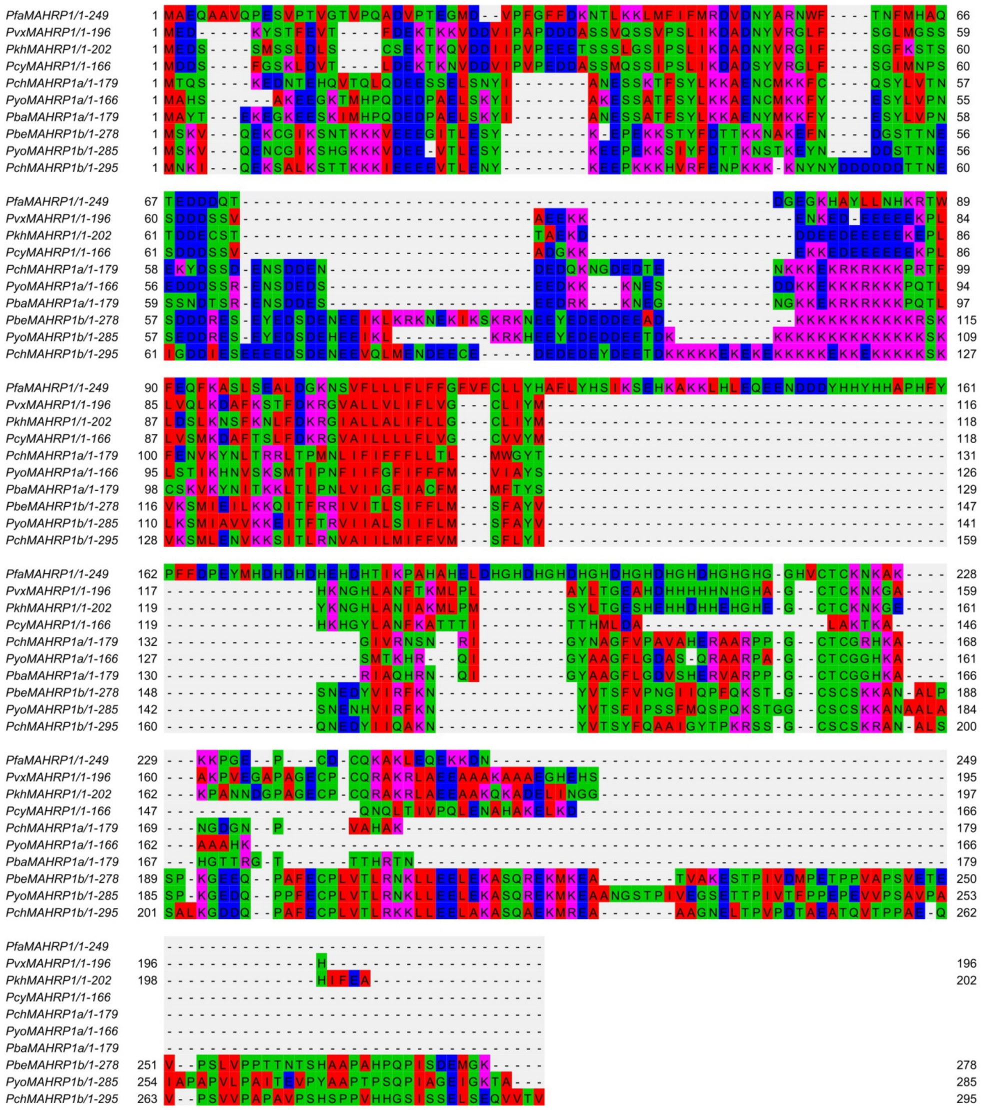

Supplementary Figure 1 | Domain structure and similarity of the identified SBP1 and MAHRP1 orthologues. (**a**), Graphical representation of domain structure of SBP1 and MAHRP1 orthologues. Scale 50 amino acids (aa). (**b**) Sequence homology tables based on the multiple amino acid alignments generated for the protein trees shown in Fig. 1c. Percentage of identical (below the diagonal) and similar amino acids (according to BLOSUM62; above the diagonal) calculated from the aligning regions are shown. Asterisk indicates that the identity/similarity scores might be biased due to incomplete amino acid sequence of PcySBP1. MAHRP1 was abbreviated to M1 in the top labels of the MAHRP table. (**c,d**) Alignments used to generate the phylogenetic trees in (a) for SBP1 (**c**) and MAHRP1 orthologues (**d**). Amino acids are highlighted as follows: A,V,L,I,M,F,W (red); G,P,C,Y,H,Q,N,S,T (green); K,R (magenta); E,D (blue).

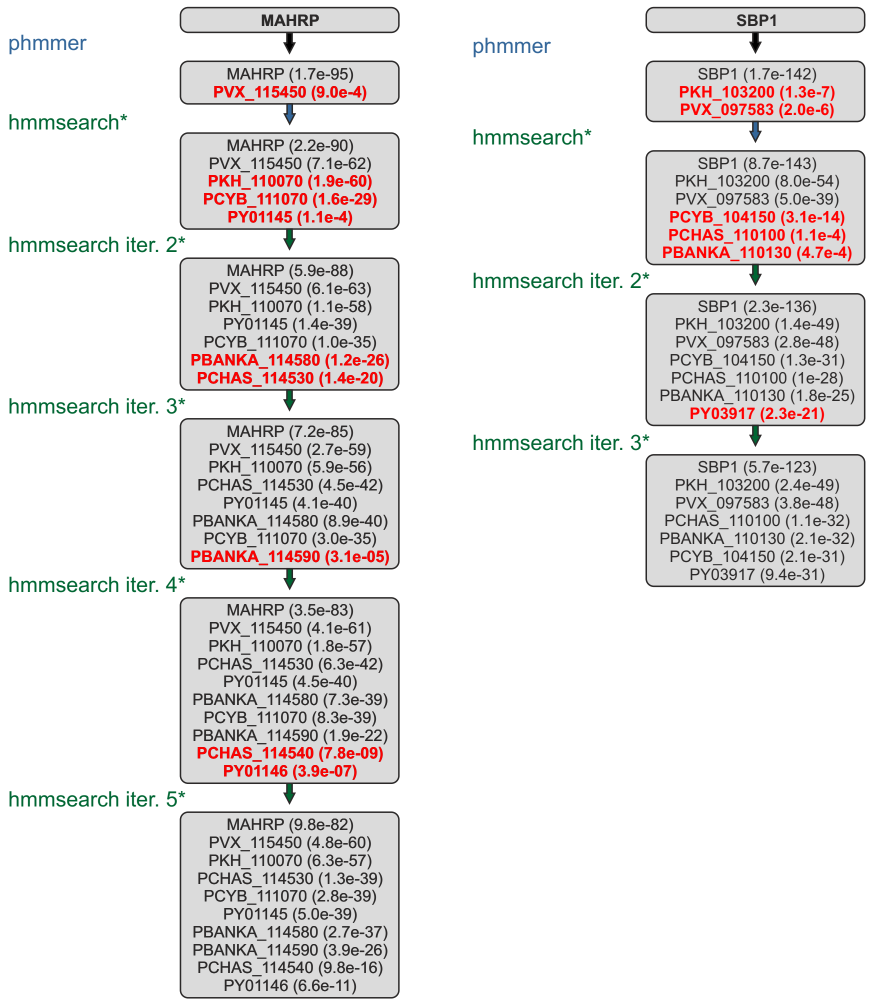

Supplementary Figure 2 | A jackhmmer search confirms the orthology of the SBP1 and MAHRP1 proteins of different *Plasmodium* species identified by BLAST searches. The search strategy is indicated on the left of the boxes; PlasmoDB gene IDs of newly identified molecules per step/iteration (iter.) are shown in red (corresponding e-values are given in brackets). An e-value inclusion threshold of 0.001 was used (indicated by an asterisk).

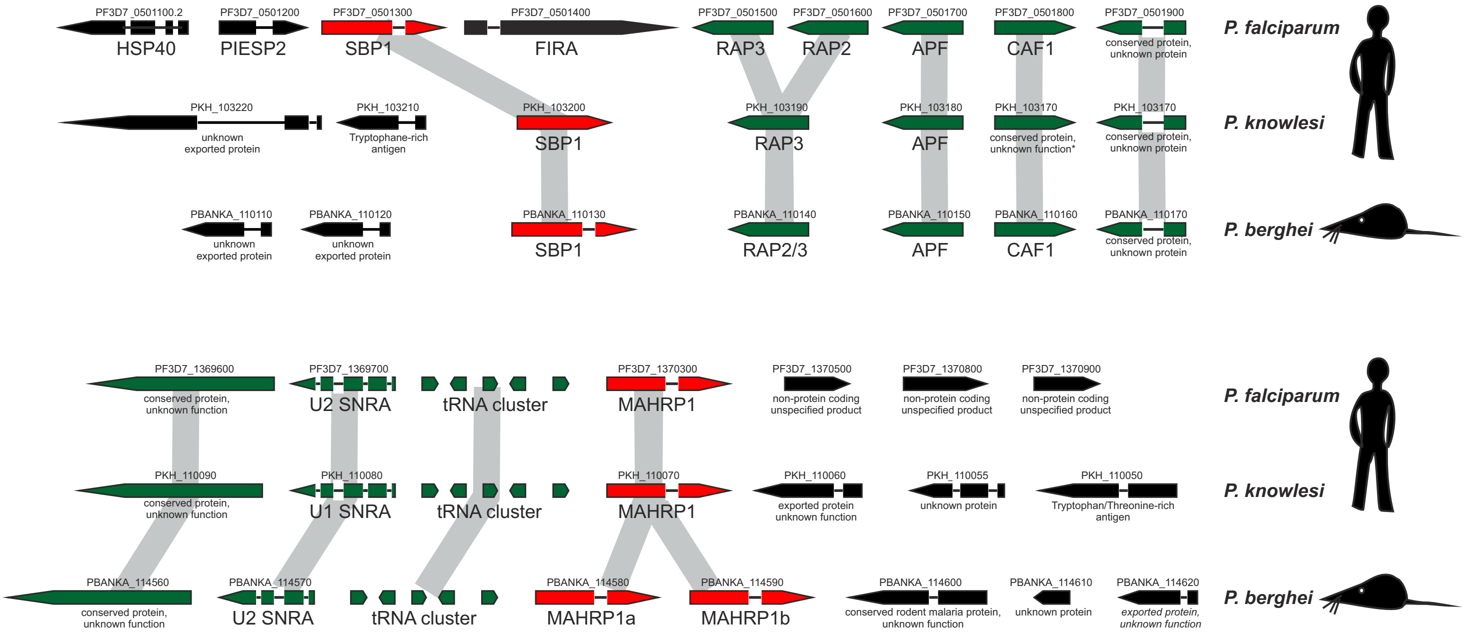

Supplementary Figure 3 | The *sbp1* and *mahrp1* genes of different *Plasmodium* species have a syntenic location in the genomes. Genomic regions harbouring the *sbp1* and *mahrp1* genes are shown for selected *Plasmodium* species. Syntenic genes with clear orthology (as defined in PlasmoDB) are shown in green, *sbp1* and *mahrp1* genes are shown in red. Synteny is indicated by gray lines. Non-syntenic genes (and genes without clear orthologues in other species) are shown in black. The size of open reading frames, introns and the intergenic regions are not shown to scale.

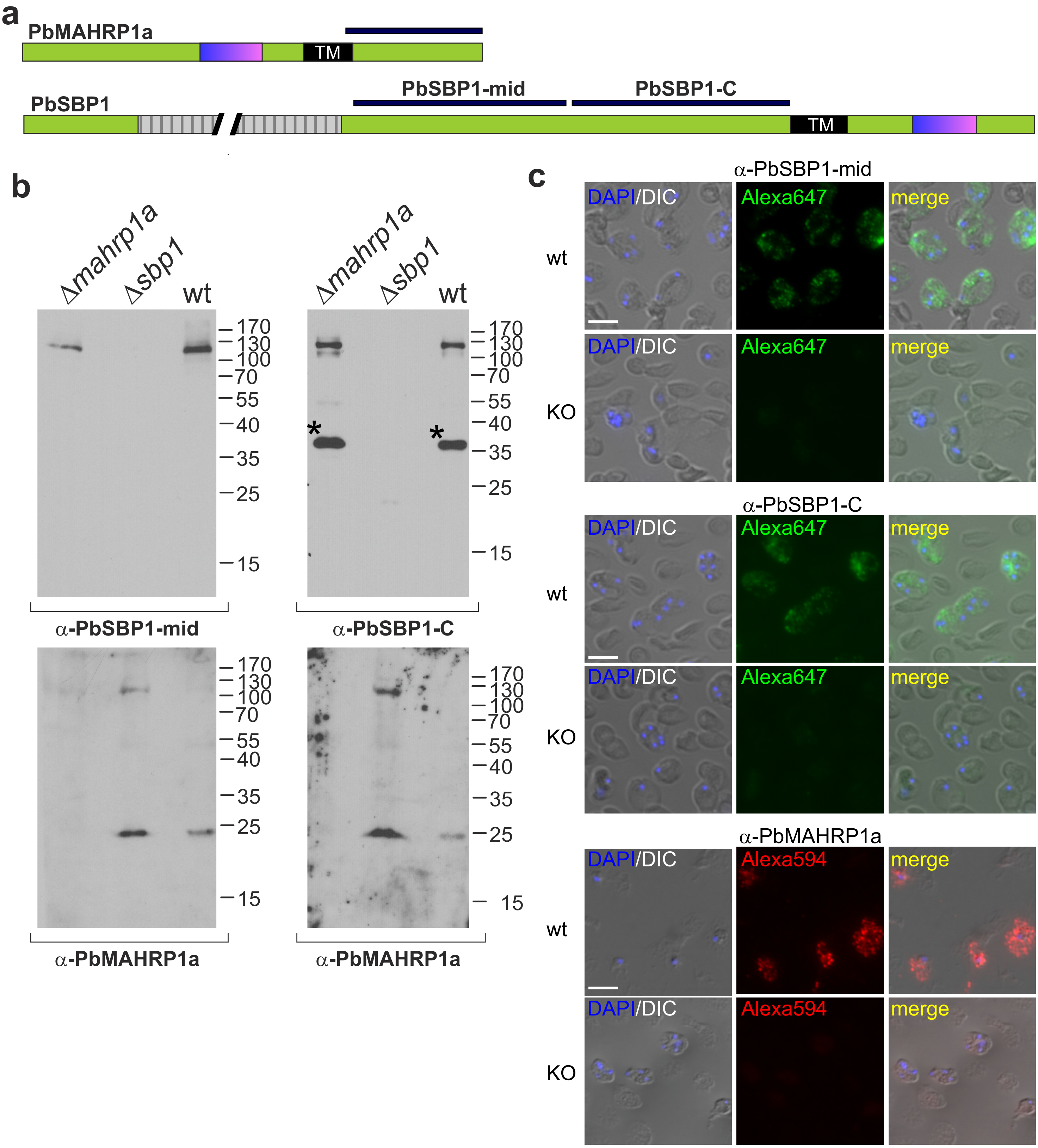

Supplementary Figure 4 | Specificity of antisera raised against PbSBP1 and PbMAHRP1a. (**a**) Schematics of PbMAHRP1a and PbSBP1 (repeat region truncated) with the regions used to raise antisera indicated as black bars. Two antisera were raised against two non-overlapping regions of PbSBP1 (PbSBP1-mid and PbSBP1-C). (**b**), Western blot analysis of *P. berghei* protein extracts from purified blood stages of wild type (wt), and PbΔ*sbp*1 and PbΔ*mahrp1a* gene-deletion parasites using the different antisera. Molecular weight standards are in kDa. The asterisk denotes a fragment detected only with α-PbSBP1-C, indicating C-terminal processing of PbSBP1. This is a PbSBP1-specific fragment, as it is not present in PbΔ*sbp1* gene-deletion parasites. The signal with α-MAHRP1a and α-PbSBP1 is used as loading control for PbΔ*sbp1* and PbΔ*mahrp1a* gene-deletion parasites, respectively. (**c**), IFA analysis of blood stages of wt and PbΔ*sbp1* and PbΔ*mahrp1a* gene-deletion parasites (KO) using the different antisera. Specific staining is only detected in wt parasites, confirming the specificity of the antisera. Merge: merge of all three channels. Size bars: 5 μm.

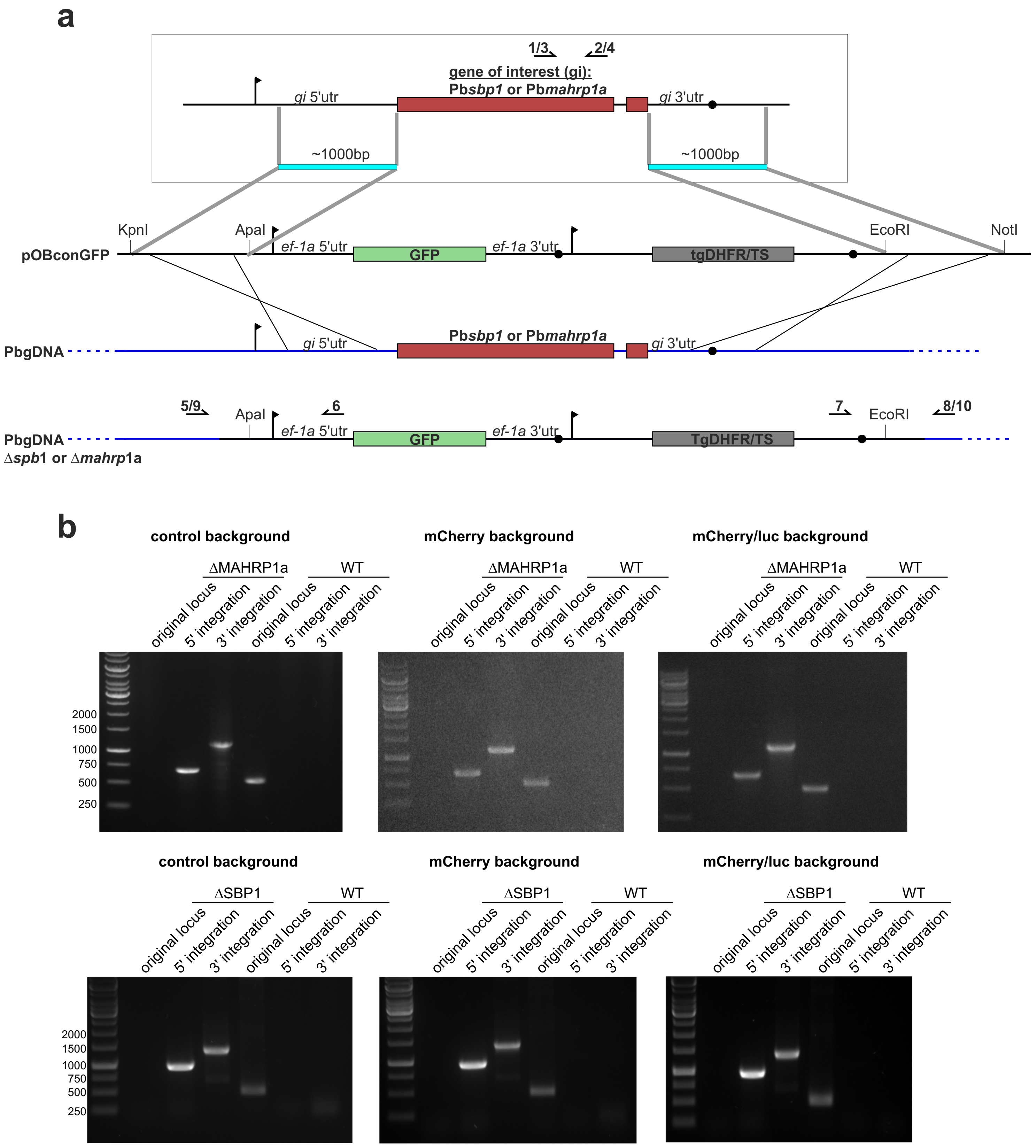

Supplementary Figure 5 | Generation of the PbΔ*sbp1* and PbΔ*mahrp1a* parasites. (**a**) The box shows a schematic of the target genes with the targeting regions inserted into the pOBconGFP gene deletion construct shown below. PbgDNA shows the unmodified genomic target region and below the genomic locus after integration of the gene deletion construct pOBconGFP. Numbered arrows: primers used in (b). (**b**) Diagnostic PCR-analysis confirm correct integration of the pOBconGFP construct in the different cloned lines of PbΔ*sbp1* and PbΔ*mahrp1a* gene-deletion parasites. Primers 1-4 were used to detect the original locus, primers 5/6 and 9/6 for 5' integration and primers 8/7 and 10/7 for 3' integration. Primers are indicated by arrows.

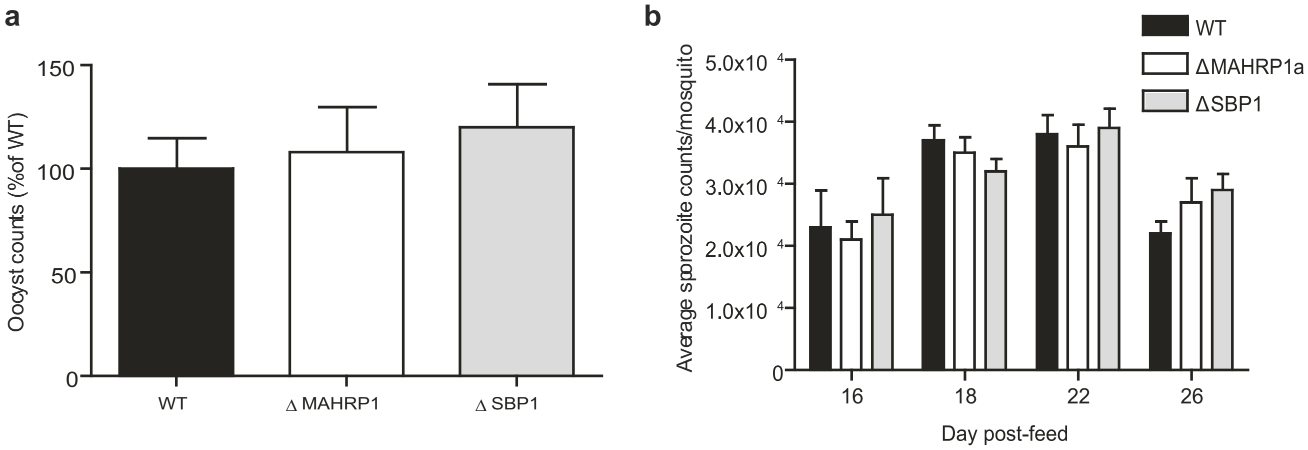

Supplementary Figure 6 | PbΔ*sbp1* and PbΔ*mahrp1a* gene-deletion parasites show development in the mosquito similar to wt parasites. (**a**) No significant difference exists in oocyst production between mosquitoes infected with wild type (wt) and gene-deletion mutant parasites (P = 0.1, Student's t-test). Oocyst numbers (n=60 mosquitoes) are shown relative to wt (%). (**b**) No significant difference in sporozoite production (numbers and timing; P = 0.08, Student's t-test) between mosquitoes infected with wt and gene-deletion mutant parasites. Salivary gland sporozoites were counted at days 16-26 post blood meal (n=60 mosquitoes).

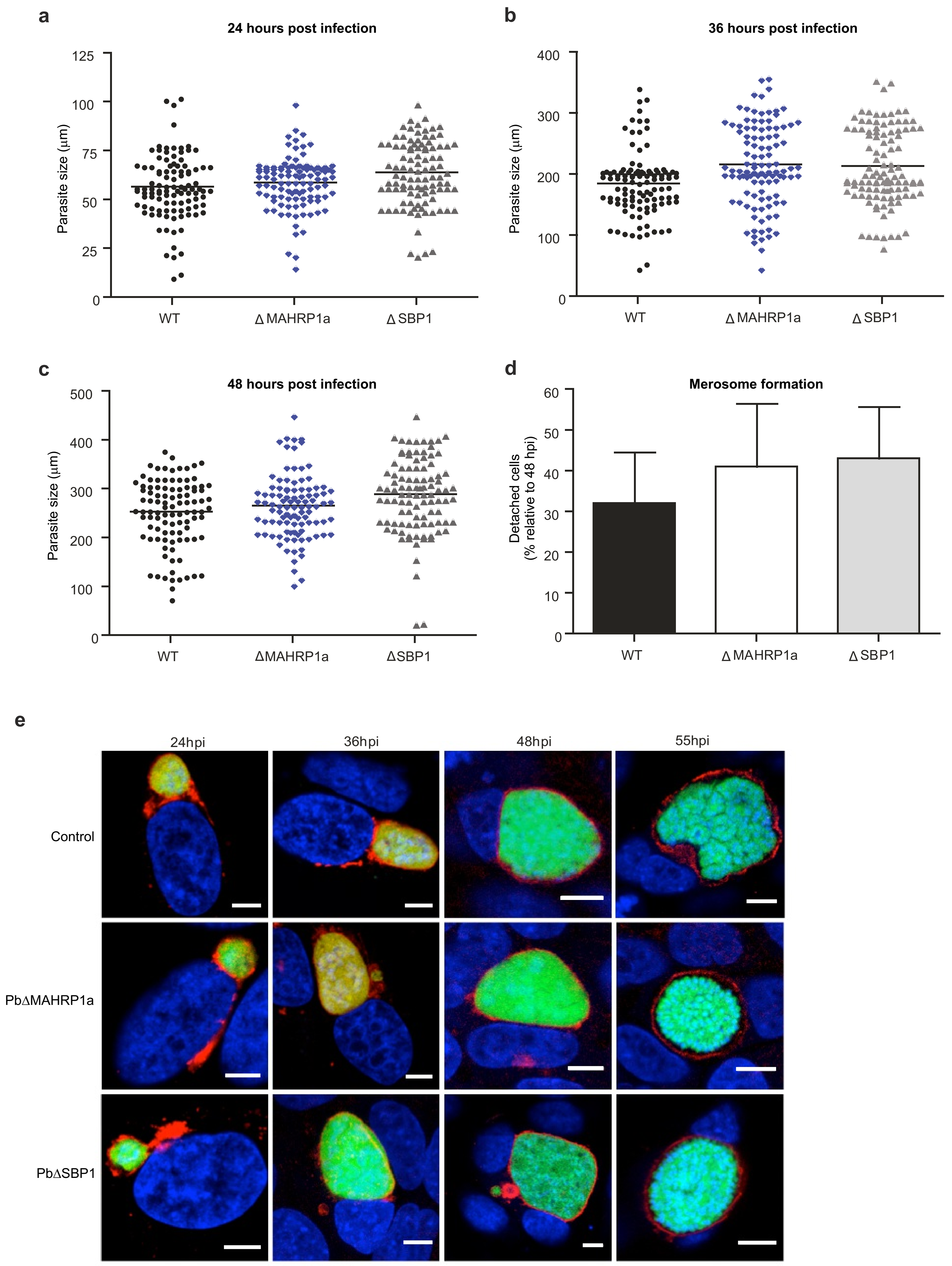
a
24 hours post infection
Parasite size (µm)
0
25
50
75
100
125
WT
Δ MAHRP1a
Δ SBP1
b
36 hours post infection
Parasite size (µm)
0
100
200
300
400
WT
Δ MAHRP1a
Δ SBP1
c
48 hours post infection
Parasite size (µm)
0
100
200
300
400
500
WT
ΔMAHRP1a
ΔSBP1
d
Merosome formation
Detached cells
(% relative to 48 hpi)
0
10
20
30
40
50
60
WT
Δ MAHRP1a
Δ SBP1
e
24hpi
36hpi
48hpi
55hpi
Control
PbΔMAHRP1a
PbΔSBP1

Supplementary Figure 7 | Pb∆*sbp1* and Pb∆*mahrp1a* parasites show development in hepatocytes similar to wt parasites. (**a-d**) Individual sizes of liver-stage parasites in cultured HeLa cells at different time points post infection (p.i.) with wt and mutant sporozoites. Sizes were measured of 100 parasites per well (triplicate wells)**.** (**d**) Merosome formation (detached cells), representing successful completion of liver stage development at 65 h p.i.. Detached cell numbers are expressed as % of parasites present at 48h p.i in the same well (triplicate wells). (**e**) Similar development of wt and mutant liver stages as visualised by confocal microscopy at various hours p.i.. Parasites were stained with the nuclear dye DAPI (blue), antibodies against the parasitophorous vacuole membrane were anti-UIS4 (red; 12-24 h); anti-exp1 (red; 36-56 h) and anti-GFP (cytoplasm; green) (GFP).

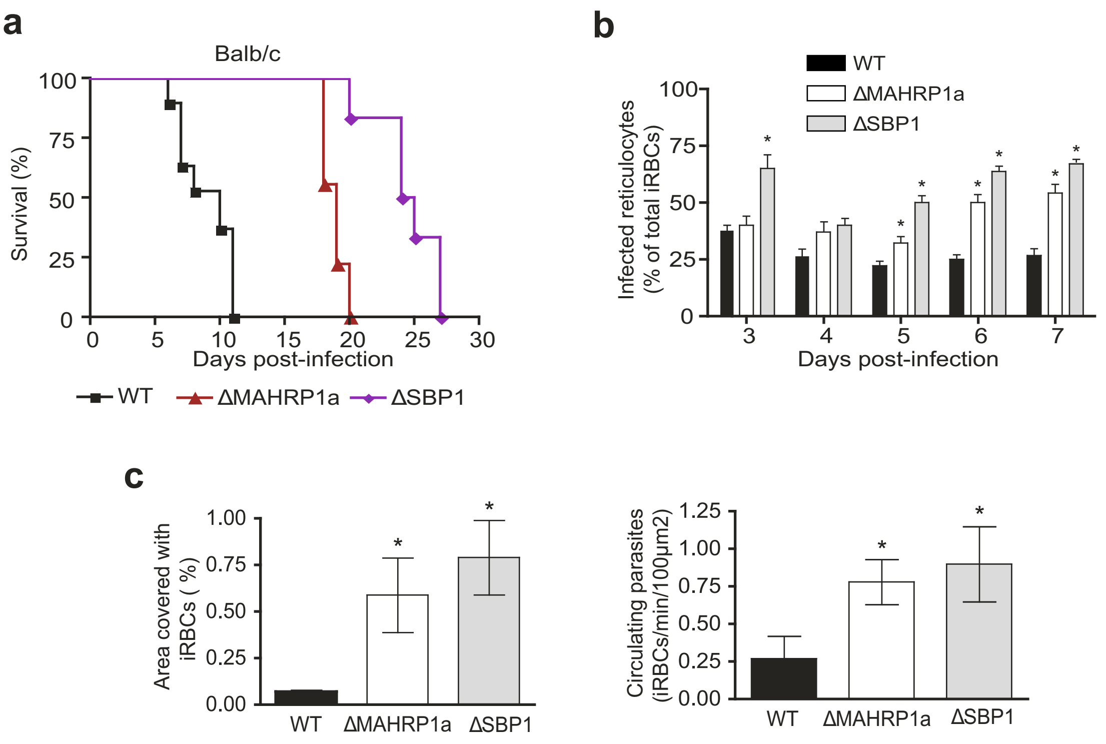

Supplementary Figure 8 | Features of blood stage infections in mice infected with wt, PbΔ*sbp1* and PbΔ*mahrp1a* parasites. (**a**) Significantly prolonged survival of Balb/c mice infected with mutant parasites compared to wt (n = 15 mice per group, logrank test p<0.001; Kaplan-Meier curves). (**b**) Reticylocyte versus normocyte preference of wt, PbΔ*sbp1* and PbΔ*mahrp1a* parasites in C57B/6 mice at days 3-7 after infection. Tropism was determined in Giemsa stained blood films. A significantly higher number of gene-deletion mutant parasites were identified within reticulocytes, as opposed to wt parasites (P <0.001, Student's t-test). **c,** Quantification of the presence of schizont-infected RBC in spleens of UBC-GFP mice at 22 hours after establishing synchronised infections of wt and mutant parasites. Fifty fields of view of splenic tissue were observed for blood flow and parasite circulation. 50 positions were selected in every spleen, and intravital movies generated for a period of 5 minutes (2 images/s). Total area covered with iRBCs adhering to the splenic tissue or circulating through splenic vasculature, was quantified. The rate of passage of circulating iRBC was measured within open circulation or vessels, and values expressed as iRBCs/ minute/ area. Experiments were performed in n = 3 mice per parasite line (P = 0.001, Student's t-test).

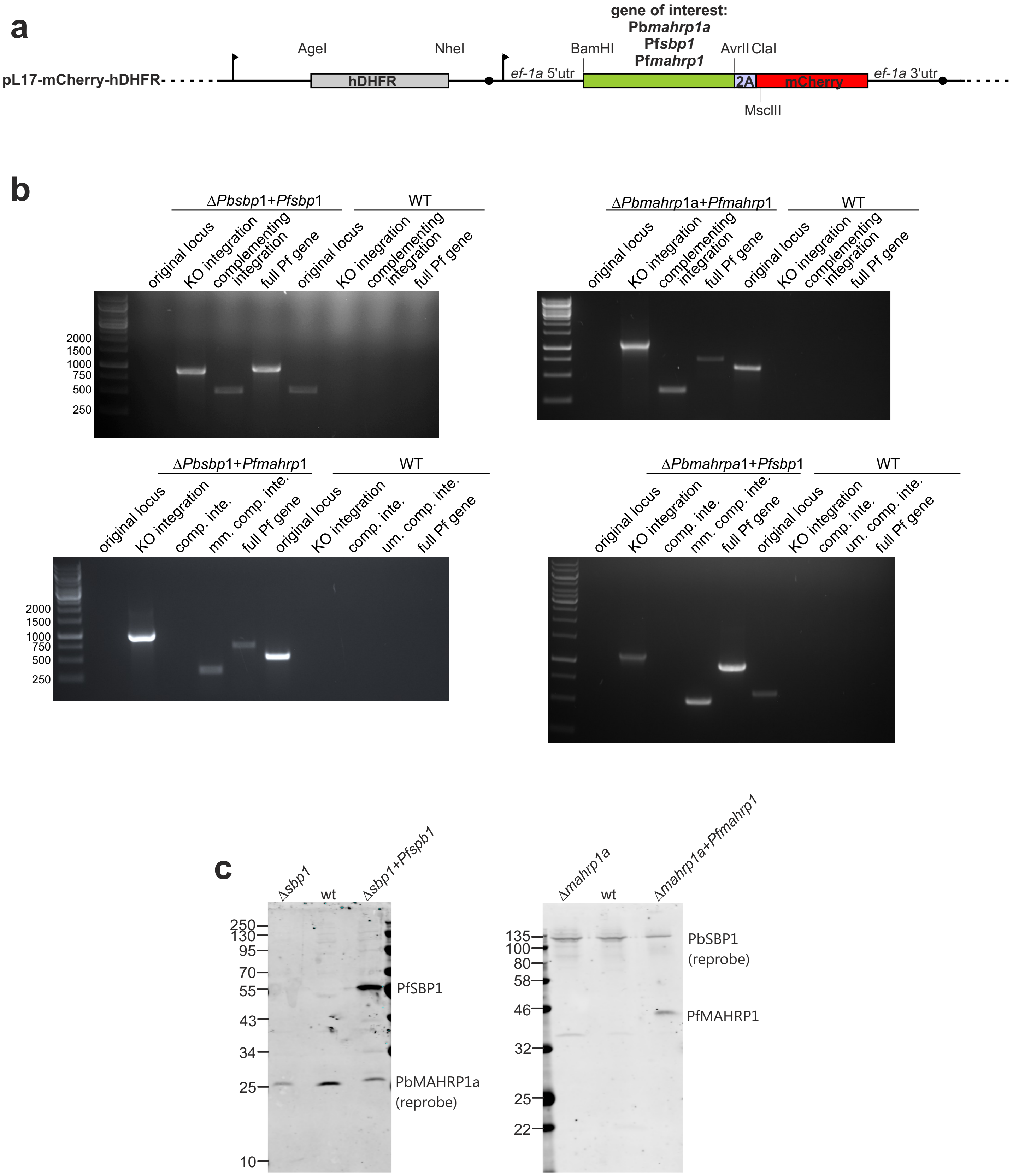
a
pL17-mCherry-hDHFR
AgeI
NheI
hDHFR
ef-1a 5'utr
BamHI
gene of interest:
Pbmahrp1a
Pfsbp1
Pfmahrp1
AvrII
ClaI
2A
mCherry
MscIII
ef-1a 3'utr
b
ΔPbsbp1+Pfsbp1
WT
original locus
KO integration
complementing integration
full Pf gene
2000
1500
1000
750
500
250
ΔPbmahrp1a+Pfmahrp1
ΔPbsbp1+Pfmahrp1
comp. inte.
mm. comp. inte.
um. comp. inte.
ΔPbmahrpa1+Pfsbp1
c
Δsbp1
wt
Δsbp1+Pfspb1
250
130
95
70
55
43
34
25
10
PfSBP1
PbMAHRP1a
(reprobe)
Δmahrp1a
Δmahrp1a+Pfmahrp1
135
100
80
58
46
32
22
PbSBP1
PfMAHRP1

Supplementary Figure 9 | Generation of the complementation of mutants. (**a**) Plasmid for the generation of complementation mutants. The part of plasmid of the pL17mCherry-hDHFR relevant for complementation is shown. Note that due to the skip peptide (2A), the complementing protein is without a tag that could affect its function while at the same time the complemented parasites express cytoplasmic mCherry. mCherry fluorescence therefore demonstrates complementation and is visible in addition to the cytoplasmic GFP fluorescence that is indicative of the respective gene deletion. mCherry and GFP fluorescence was microscopically confirmed for all clones used. (**b**) Diagnostic PCR-analysis confirms correct integration of the matched and mismatched complementation constructs in the cloned PbΔ*sbp1* and PbΔ*mahrp1a* gene-deletion parasites. 'Comp. inte.', complementing integration; 'mm. comp. inte.', mismatched complementing integration; 'full Pf gene', detection of full complementing gene. To confirm integration, primers spanning the junction between the insertion and the 5' genomic region were used (primer AB_ef1a 5' UTR - Fw with either PfMAHRP1_int_rev or PfSBP1_int_rev, see Table S1). To confirm the presence of the full gene, primers binding at the 5' and 3' end of each gene were used (primers PfMAHRP1_full_fw with PfMAHRP1_full_rev and PfSBP1_full_fw with PfSBP1_full_rev, see Table S1). (**c**) Western blot showing expression of the complementing *P. falciparum* proteins in the gene deletion parasites but not uncomplemented or wt parasites. As a loading control PbMAHRP1a was detected in the PbΔ*sbp1* parasites and PbSBP1 in the PbΔ*mahrp1a* parasites (by re-probing onto the same membrane).

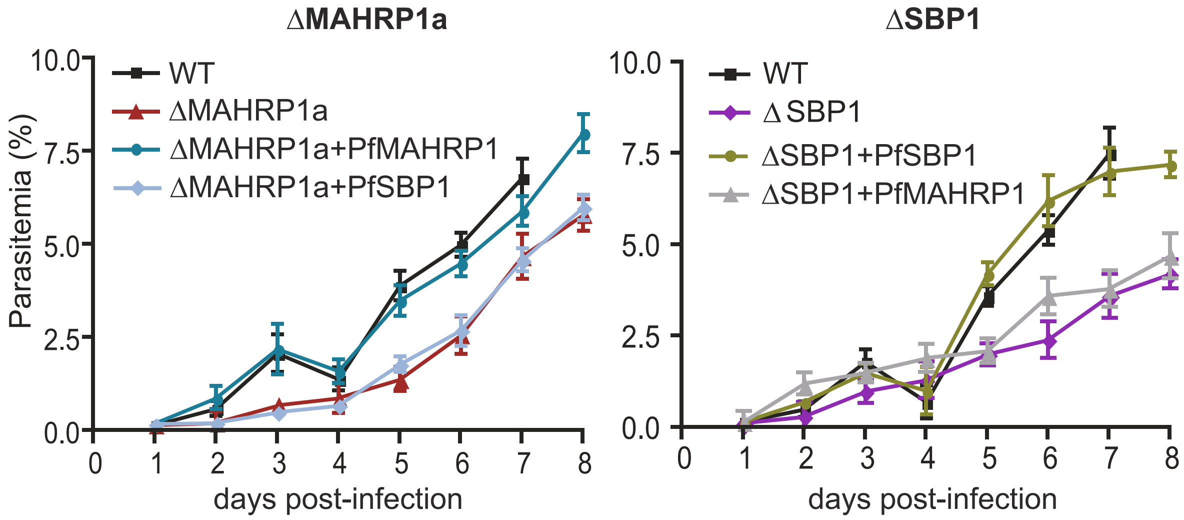

Supplementary Figure 10 | The course of parasitemia in C57BL/6 mice infected with wt, mutant and complemented mutants. Complementation with Pf*mahrp1* and Pf*sbp1* restore a growth rate similar to that of wt *P. berghei* (n=12 mice per complemented mutant). The parasitemia of matched complemented parasites was similar to the parasitemia of wt parasites. The parasitemia of mismatched complemented parasites was similar to that of the gene-deletion mutants (*P*=0.07, Student's *t*-test).

Supplementary Table S1: Primers used in this study

Primer target	Primer sequence 5' - 3'	Purpose
PbSBP1-5utr-Kpnfw2	CAGCGGTACCGTTATATGTATCTTCTAAAAGGATAGAAGC	PbSBP1 KO construct
PbSBP1-5´utr-Aparv2	GCTGGGGCCCGCAATTGATTCAGGAGCATAAGTTGATATTTGATTATC	PbSBP1 KO construct
PbSBP1-3´utr-EcorIfw	CAGCGAATTCGGATATAAAATTATAAAAAGTTATTTAATGTAAAG	PbSBP1 KO construct
PbSBP1-3´utr-Notrv	GCTGGCGGCCGCCCGAATTAATCTTCTTAAGAGAAAGTTTTTTTGATGC	PbSBP1 KO construct
hDHFR-F-AgeI	ATACCGGTAATGGTTGGTTCGCTAAACTGC	AB plasmid cloning
hDHFR-R-NheI	ATGCTAGCATTAATCATTCTTCTCATATACTTCAAATTTGTA	AB plasmid cloning
PbMAHRP1a_fw_BamHI	CAGCGGATCCTAAAATGGCTTACACTGAAAAAGAAGGTAAAGAAG	PbMAHRP1a AB construct
PbMAHRP1a_rev_AvrII_part 2A_1	GGTCCTGGATTTTCTTCTACATCTCCACATGTTAATAAACTTCCTCTTCCTTCTCCCCTAGGTCAGTTAGTTCTATGGGTAGTAGTGCCTCTAGTAGTACC	PbMAHRP1a AB construct
PbMAHRP1a_rev_ClaI_part-2A_part mCherry_MscI_2	GCTCTGGCCATGTTATCCTCCTCGCCCTTGCTCACCATATCGATTGGTCCTGGATTTTCTTCTACATCTCC	PbMAHRP1a AB construct
PfMAHRP1_fw_BamHI	CAGCGGATCCTAAAATGGCAGAGCAAGCAGCAGTACAACCAGAAAG	PfMAHRP1 AB cloning
PfMAHRP1_rev_AvrII	GCTCCCTAGGATTATCTTTTTTTTCTTGTTCTAATTTTGC	PfMAHRP1 AB cloning
PfSBP1_fw_BamHI	CAGCGGATCCTAAAATGTGTAGCGCAGCTCGAGCATTTG	PfSBP1 AB cloning
PfSBP1_rev_AvrII	GCTCCCTAGGGGTTTCTCTAGCAACTGTTTTTGTTGTGGATTTGG	PfSBP1 AB cloning
PbMAHRP1a_C_fw_BamHI	CAGCGGATCCACATATTCAAGGATTGCACAGCATAGAAATC	PbMAHRP1a pGEX cloning
PbMAHRP1a_C_rev_XhoI	GTCGCTCGAGTCAGTTAGTTCTATGGGTAGTAGTGCCTCT	PbMAHRP1a pGEX cloning
PbSBP1_mid_fw_BamHI	CAGCGGATCCCAAGATACAGAAAATAAATTTCAAGATACAGAAAAGAAATTTTTAGATACAGAAAAGAAATTTCAAGATAAAGAAAAGAAATATCTC	PbSBP1 pGEX cloning
PbSBP1_mid_rev_XhoI	GTCGCTCGAGATAAGGAGAATCAATTGCAGTTTTTACATTAGAACCTGGACTATCATCTCCAAGAATTGGTATTGTATTGGTTTCAGATAATTTTGG	PbSBP1 pGEX cloning
PbSBP1_C_fw_BamHI	CAGCGGATCCAATGCTCAACCGCCAACTAGTATTCAAAGTG	PbSBP1 pGEX cloning
PbSBP1_C_rev_XhoI	GTCGCTCGAGTATTAAACCTTTTCCGTGAAATATTCTTCC	PbSBP1 pGEX cloning
PbSBP1-bam-fw	CAGCGGATCCAAAAATGGATAATCAAATATCAACTTATGCTCC	PbSBP1 pL17 cloning
PbSBP1-bam-rv	CAGCGGATCCTTTTTTTTTAAATTTCTTTTCGGTCTTATTTGCC	PbSBP1 pL17 cloning
KpnI-PbMAHRP1a1-20-mTRAP_F	CAGCGGTACCATGGCTTACACTGAAAAAGAAGGTAAAGAAGAAAGTAAAATTATGCATCCCCAAGATGAATCTGCATTATATGAACATATGAATAC	mTRAP fusion cloning
KpnI-PbSBP1-1-20-mTRAP_F	CAGCGGTACCATGGATAATCAAATATCAACTTATGCTCCTGAATCAATTGCTTCAAATATATTATTAGATTCTGCATTATATGAACATATGAATAC	mTRAP fusion cloning
	CGCGCCTAGGTTCGAGTGCCCAGAATTCTTCTTC	
PbMAHRP1a- Fw (1)*	CGAGAGTGAAGAAGATAG	Diagnostic PCR WT MAHRP1a
PbMAHRP1a- Rev (2)*	ATTTTTAGGTGATGTAAGTCATGAACG	Diagnostic PCR WT MAHRP1a
PbSBP1- Fw (3)*	GAAGCAGCACAAGCGGAACAAGT	Diagnostic PCR WT SBP1
PbSBP1- Rev (4)*	GCAAGTAAACATAACAAAAAGGCC	Diagnostic PCR WT SBP1

PbΔMAHRP1a 5’ Fw (5)*	GTCCTGAAACTAATATAAAGGGCC	Diagnostic PCR MAHRP1aKO
ef1a 5’ UTR - Rev (6)*	TTATTTGCACTACTGGAAAACTACC	Diagnostic PCR KO
Tgdhfr 3’- Fw (7)*	TAGCGGAAATACAGAAGCTAGC	Diagnostic PCR KO
PbΔMAHRP1a 3’ Rev (8)*	GCATAATTTTTCTGAACCATCTGG	Diagnostic PCR MAHRP1a KO
PbΔSBP1 5’ Fw (9)*	AAAAATACAATCTCTAAGGAGGTGC	Diagnostic PCR SBP1 KO
PbΔSBP1 3’ Rev (10)*	TGCGCCGACCATCTTAGTATC	Diagnostic PCR SBP1KO
PfMAHRP1_full_fw	ATGGCAGAGCAAGCAGCAGTACAACCAGAAAG	Diagnostic PCR for full PfMAHRP1
PfMAHRP1_full_rev	ATTATCTTTTTTTTCTTGTTCTAATTTTGC	Diagnostic PCR for full PfMAHRP1
PfSBP1_full_fw	ATGTGTAGCGCAGCTCGAGCATTTG	Diagnostic PCR for full PfSBP1
PfSBP1_full_rev	GGTTTCTCTAGCAACTGTTTTTGTTGTGGATTTGG	Diagnostic PCR for full PfSBP1
AB_ef1a 5’ UTR - Fw	GTTGTGAAACAAAAAACG	Diagnostic PCR for AB
PfMAHRP1_int_rev	CATCAACTTCTTTAAAG	Diagnostic PCR for PfMAHRP1AB
PfSBP1_int_rev	GTGCCTCTGCTGCATCACCAACTAAATTTCC	Diagnostic PCR for PfSBP1AB

*number refers to the primers indicated in Extended Data Figure 5
